# Design of dual mode antenna using CMA and broadband dual-polarized antenna for 5G networks

**DOI:** 10.1038/s41598-024-66515-x

**Published:** 2024-07-05

**Authors:** N. Sathishkumar, SatheeshKumar Palanisamy, Rajesh Natarajan, Khmaies Ouahada, Habib Hamam

**Affiliations:** 1 Department of Electronics and Communication Engineering, KPR Institute of Engineering and Technology, Coimbatore, Tamil Nadu 641407 India; 2 Department of Electronics and Communication Engineering, BMS Institute of Technology & Management, Yelahanka, Bengaluru, 560064 Karnataka India; 3 School of Electronics Engineering, Vellore Institute of Technology, Vellore, Tamil Nadu 632014 India; 4 School of Electrical Engineering, University of Johannesburg, Johannesburg, 2006 South Africa; 5 Faculty of Engineering, Université de Moncton, Moncton, NB E1A3E9 Canada; 6 Hodmas University College, Taleh Area, Mogadishu, Somalia; 7 Bridges for Academic Excellence, Tunis, Tunisia

**Keywords:** Dual mode antenna, Dual band antenna, Dual polarized antenna, Vertical polarization, Horizontal polarization, CST, Mathematics and computing, Engineering, Electrical and electronic engineering

## Abstract

This article proposes a dual mode dual-polarized antenna configuration for IRNSS and fifth generation (5G) applications, operating at a frequency of 3.5 GHz based on characteristic mode analysis (CMA), and aims to provide broadband dual-polarized functionality. The original design of the antenna is a traditional patch antenna, and its dual-polarized features are determined using characteristic mode analysis. The full-wave method is used to stimulate both orthogonal modes using a 50 Ω coaxial input line at 3.5 GHz. In this design, the circular patch has been extended into an elliptical patch through a process of mode separation. The circular patch exhibits resonance at a frequency of 2.5 GHz, whereas the extended elliptical radiator demonstrates two resonance modes at 2.5 GHz and 3.5 GHz. The operational mechanism is elucidated by modal analysis and characteristic angle. This antenna operates on two different frequencies at the 2.5 GHz IRNSS band with horizontal polarization and the 3.5 GHz 5G service with vertical polarization. The maximum gain achieved with these frequency ranges is 5.31 dBi and 4.72 dBi, respectively. A ring resonator is chosen to improve the axial ratio and impedance bandwidth of the suggested prototype. The antenna's ground plane is shaped like a rectangle and features a V-shaped slot in the radiating patch. The antenna's physical footprint is 50 mm × 50 mm × 1.6 mm and an FR4 dielectric substrate serves as its foundation. Through its interaction with a PIN diode, the diode modifies the polarization of the antenna. The antenna functions as a right-handed circular polarization (RHCP), when the diode is operational. The bandwidth from 4.3 to 7.5 GHz is covered. On the other hand, it generates linear polarization (LP) between 4.2 and 5.3 GHz. The experimental antenna is evaluated and examined for its performance characteristics. The simulations are carried out utilizing the CST simulator. A prototype antenna has been manufactured and its performance has been validated against simulated findings.

## Introduction

### Context and motivation

The relentless demand for higher data rates, increased capacity, and seamless connectivity has propelled the evolution of wireless communication technologies toward the advent of 5G networks. As 5G deployment progresses, the development of efficient and versatile antenna systems becomes imperative to meet the diverse requirements of emerging applications. Antennas are the backbone of wireless communication systems, dictating their performance and capabilities. In the context of 5G, the design of antennas must address challenges such as increased data rates, massive connectivity, and compatibility with diverse frequency bands. This research focuses on the design and optimization of two innovative antenna solutions tailored for 5G networks: a dual-mode antenna utilizing Conformal Mapping Approach (CMA) and a broadband dual-polarized antenna. Both designs aim to address the evolving needs of 5G communication, including enhanced spectral efficiency, improved coverage, and seamless integration with existing infrastructure. The dual-mode antenna leveraging CMA offers a unique approach to achieve multi-functionality within a compact form factor. By harnessing the principles of conformal mapping, this antenna design promises versatility in frequency tuning and radiation pattern shaping, crucial for accommodating the diverse frequency bands and beamforming requirements of 5G systems.

In parallel, the broadband dual-polarized antenna presents a novel solution to the challenges of polarization diversity and spectral efficiency in 5G networks. By enabling simultaneous transmission and reception of orthogonal polarizations over a wide frequency range, this antenna design enhances system capacity and mitigates polarization-related fading effects, thereby improving overall network reliability and performance. Through comprehensive simulation and experimental validation, this research aims to demonstrate the efficacy and practicality of the proposed antenna designs for 5G networks. Performance metrics such as radiation efficiency, impedance matching, and cross-polarization discrimination will be evaluated to ascertain the suitability of the antennas for real-world deployment scenarios. In summary, the design of dual-mode antenna using CMA and broadband dual-polarized antenna represents a significant step towards realizing the full potential of 5G communication. By providing versatile, efficient, and reliable antenna solutions, this research contributes to the ongoing evolution of wireless networks, paving the way for a connected future characterized by seamless connectivity and ubiquitous access to high-speed data services.

### Key aspects and novel elements

The main contributions of the authors may be summarized as follows.

- Dual Mode Antenna Design using Characteristic Mode Analysis (CMA): Introduced a unique dual-mode, dual-polarized antenna design that leverages characteristic mode analysis (CMA) to separate dominant TM10 and TM11 modes. By reshaping a circular patch into an elliptical one, the design achieves dual resonance at 2.5 GHz (IRNSS) and 3.5 GHz (5G) with distinct horizontal and vertical polarization modes.
- Broadband Dual-Polarized Antenna for 5G Networks: Proposed a novel broadband dual-polarized antenna that utilizes a ring resonator and a PIN diode to switch between linear polarization (LP) and right-hand circular polarization (RHCP). The antenna covers a wide frequency range from 4.3 to 7.5 GHz (RHCP) and 4.2–5.3 GHz (LP), providing enhanced axial ratio and impedance bandwidth.
- V-Shaped Slot for Polarization Diversity and Enhanced Bandwidth: Incorporated a V-shaped slot in the antenna's ground plane to achieve polarization diversity and improve axial ratio and impedance bandwidth. This innovative slot design ensures equal amplitude and 90° phase difference in currents, resulting in circular polarization over a broad frequency range.
- Comprehensive Experimental Validation and Practical Implementation: Fabricated and validated prototypes for both antenna designs through simulation and experimental measurements, demonstrating excellent correlation between simulated and measured results. Comprehensive evaluation of performance metrics like radiation efficiency, impedance matching, and cross-polarization discrimination confirms their suitability for 5G network deployment.

### Structure of the article

Sections “Introduction” and “Literature review” presents a concise summary of the structure and arrangement of the paper. Section “Dual mode antenna design using CMA” delivers a comprehensive overview of the fundamental design, analytical, and CMA investigations conducted on the dual-mode antenna. In Sect. “Broadband dual-polarized antenna geometry”, we discussed the optimization of the feed for the original antenna, which has broadband dual-polarized characteristics. To enhance connectivity and improve signal strength, Sect. “Conclusion” introduces the validation of the suggested antenna. Section “Broadband dual-polarized antenna geometry” gives the final findings and conclusions of the paper.

## Literature review

### Current research

The advancement of current RF front-end technologies has led to an increase in the need for dual-mode dual-polarized antennas in smaller spaces. The dual-polarized antennas have acquired considerable interest because of their compact dimensions, lightweight, affordability, and seamless integration with circuits ^1–3^. The rising ubiquity of electronic devices that support wireless connectivity has led to an escalating demand for swift data transmission, especially in critical sectors like radar, object monitoring, and multimedia applications ^4–6^. The CMA (Characteristic Mode Analysis) is a fundamental method used in antenna design to assess resonance in free space and analyze antenna performance. It comprises the calculation of the radiated field and current distribution. The antenna is a crucial element of navigation technology, as it determines the efficiency of device functionality ^7–9^. A comprehensive evaluation of the antenna design is essential for navigation technology to guarantee optimal system performance. Due to their low-profile design and simple integration with GNSS technology, micro strip antennas have attracted considerable interest in recent decades ^10–12^. They are especially suitable for use in tiny navigation systems. The positioning and measurement services that are precise in real time have become crucial in modern communication systems. The IRNSS is a self-governing regional navigation system that offers precise positioning services. The fabricated device can function in two frequency bands, with a central resonance of 1.176 GHz and 2.492 GHz ^13^. The commercial deployment of fifth-generation (5G) communications is currently underway in several places worldwide. It is reported that 5G has significantly improved speed, and coverage, and resolved latency and congestion issues that were present in 4G networks. A patch antenna operating at a frequency of 2.49 GHz for IRNSS is developed ^14–16^. The proposed device has a gain of 4.6 dBi and a directivity of 6.9 dB. The suggested microstrip patch antenna exhibits a return loss of − 41 dB and has a bandwidth of 71.7 MHz, with a VSWR of approximately 1.01. A small and affordable microstrip patch antenna specifically for S-band use in IRNSS receivers is investigated ^17,18^. A highly efficient wireless system is the navigation system. The United States administration exercises authority over GPS, a widely recognized navigation system. IRNSS is a novel satellite-based positioning and navigational system designed by the ISRO. It is examined that the operating frequencies of this system are 2492.028 MHz and 1176.45 MHz in the L-band. This study addressed the positioning error using the tri-band antenna, which operates on L_1_, L_5_, and S-band frequencies ^19,20^. The GPS antenna demonstrated the lowest attitude error. The increasing significance of ultra-wideband (UWB) applications is driven by the expansion of cellular, satellite, and wireless modes of communication ^21^. Consequently, there is a growing need for wideband or multiband antennas with circular polarization. The dual-band option is more advantageous for any navigation satellite system due to the impact of the ionosphere on frequency signals. The ionosphere's dispersive features resulted in a frequency-dependent phase shift. The solution to this issue can be achieved by the utilization of dual frequency, which further provides other benefits such as redundancy and enhanced immunity to interference ^22^. Creating a multi-band antenna that is both effective and compact, with easy fabrication and smooth integration with other circuit components and feed networks, while operating in many assigned frequency bands, is a difficult issue. It is understood that multi-band antennas are typically developed utilizing common patch shapes. It is feasible to achieve a compact multi-band microstrip patch antenna with good radiation performance by using a circular or elliptical shape instead of other patch shapes ^23^.

The suggested micro strip antennas are well-suited for use in aviation and mobile applications because they have a compact design, are lightweight, and have a restricted power handling capacity. They can be developed in many configurations to offer enhanced gain and frequency range, including dual-band and ultra-wideband capabilities. Wireless service providers have considered implementing polarization diversity and frequency diversity strategies to optimize the use of the restricted number of frequency bands available for communication ^24^. (Dual Band Dual Polarized) DBDP antennas have successfully fulfilled the requirements of multiple devices in wireless communication networks. The author suggests the use of an indoor mobile base station equipped with a dual-polarized antenna capable of operating in many frequency bands, including 2G, 3G, 4G, and 5G. A suggestion was made to introduce 5G mobile communication using a dual-band, 4-antenna planar MIMO setup that incorporated an L-shaped meta-rim for each folding monopole antenna, SAR is within acceptable limits and can be used for 5G mobile phone applications. The investigated antenna has dual resonances, enabling it to operate at two different frequencies 1.6–3.6 GHz and 4.1–6.1 GHz. When selecting antennas for the next 5G smartphones, it is important to prioritize those with high gain and efficiency. Different configurations of dual-band dual polarization micro strip antennas (MSA) at the S and X bands have been reported ^25^. The dual polarization has been achieved by utilizing directly coupled micro strip lines, coaxial feeds, acoustic coupling, and electromagnetic coupling (EMCP) through probe-feeding and gap-feeding methods.

The objective of this design is to develop a dual-polarized antenna for IRNSS applications, applying a simple reconfigurable method to modify the polarization. Additionally, a broadband dual-polarized antenna for 5G wireless networks will also designed.

### Research gap

Despite significant advancements in dual-mode and dual-polarized antenna technologies, several critical gaps persist, necessitating further exploration.

- Limited Application of Characteristic Mode Analysis (CMA): While CMA has been recognized as a valuable tool in antenna design, its application to achieve dual-mode dual-polarized antennas remains underexplored. Existing designs lack comprehensive analysis of modal significance and characteristic angles to harness the full potential of mode separation and achieve versatile multi-band, dual-polarized functionality.
- Inadequate Polarization Reconfigurability in Broadband Antennas: Current broadband dual-polarized antennas often fail to deliver reliable polarization reconfigurability. Many designs either lack the flexibility to switch between linear and circular polarization or suffer from poor impedance matching across the broadband range. Thus, a need exists for a more efficient mechanism to switch between polarization states while maintaining impedance bandwidth.
- Suboptimal Polarization Diversity Techniques: Conventional designs rarely incorporate innovative slot geometries to enhance polarization diversity. The absence of effective polarization diversity techniques leads to inconsistent axial ratio and polarization performance, hindering the full exploitation of polarization diversity in real-world applications.
- Limited Practical Validation of Prototype Antennas: Many proposed antenna designs lack rigorous experimental validation through fabricated prototypes. The discrepancy between simulation and experimental results often arises due to fabrication inaccuracies, leading to unmet performance expectations in practical deployment scenarios.

These gaps underscore the necessity for the development of versatile antenna designs with enhanced dual-mode, dual-polarized functionality, efficient polarization reconfigurability, and comprehensive experimental validation, which are addressed in this work.

## Dual mode antenna design using CMA

**Figure 1:**
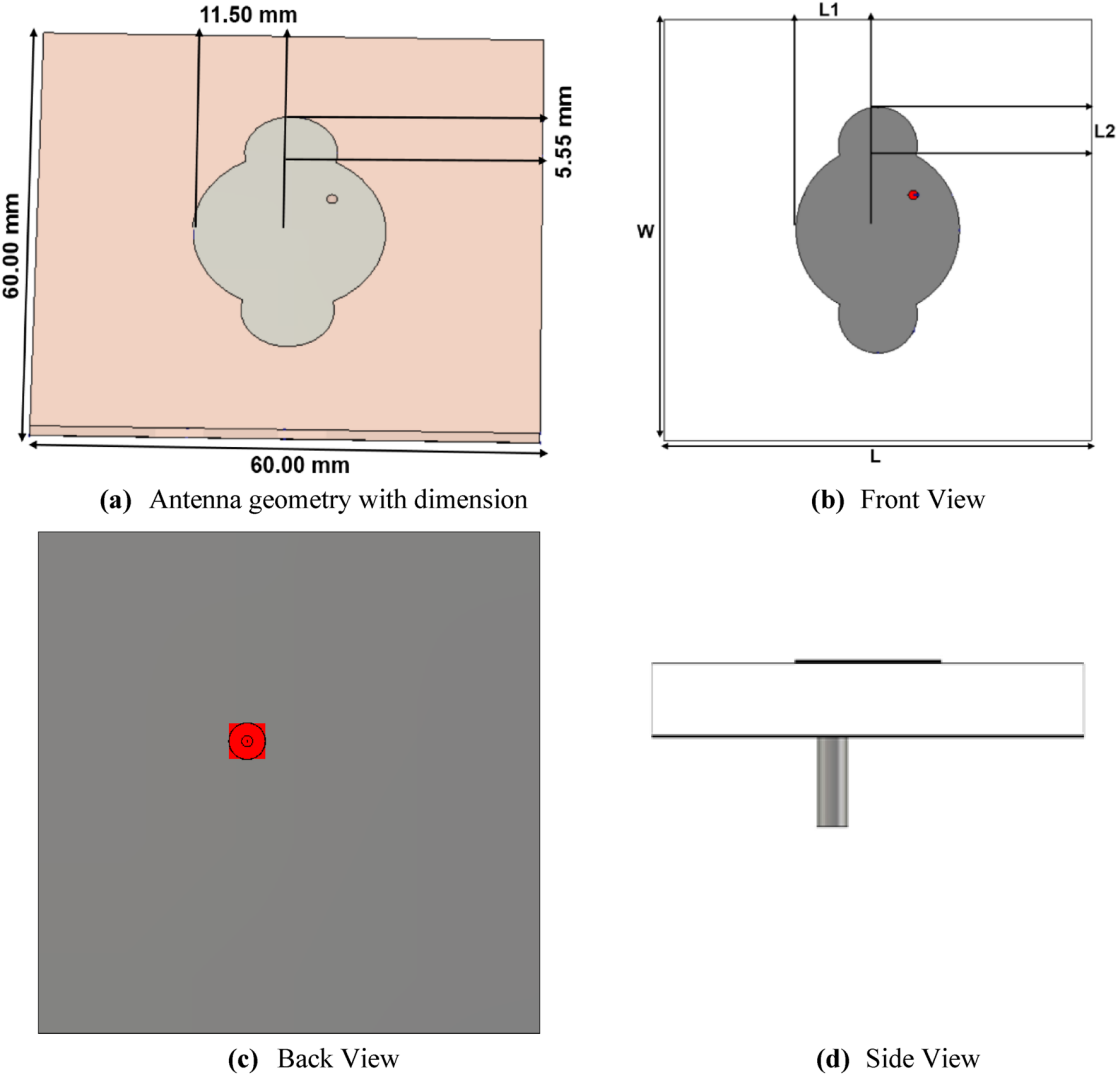
Suggested antenna structure.

The fundamental design of the suggested patch antenna begins by exciting the dominant TM_10_ mode, using the cavity model stated in Eqs. (1–6). The traditional circular patch antenna is utilized to generate the primary frequency of 2.5 GHz. To reach a frequency of 3.5 GHz, the radiating patch is reshaped into an extended elliptical shape, and the dominant mode TM_11_ is separated into its orthogonal mode components. Figure 1 depicts a systematic design of the recommended antenna shape utilizing the cavity model theory.

The FR-4 substrate is utilized to print the suggested antenna geometry, which is illustrated in Fig. 1, and has the following physical dimensions of 60 mm × 60 mm × 1.6 mm. Before transforming into an extended elliptical-shaped radiator, a circular patch antenna is initially devised to calculate the number of dominant modes produced on the radiator. The circular radiator has a radius of 11.50 mm. Two circles are appended to the circular radiator with a radius of 5.55 mm along its horizontal axis to make it an extended elliptical-shaped radiator.

**Figure 2:**
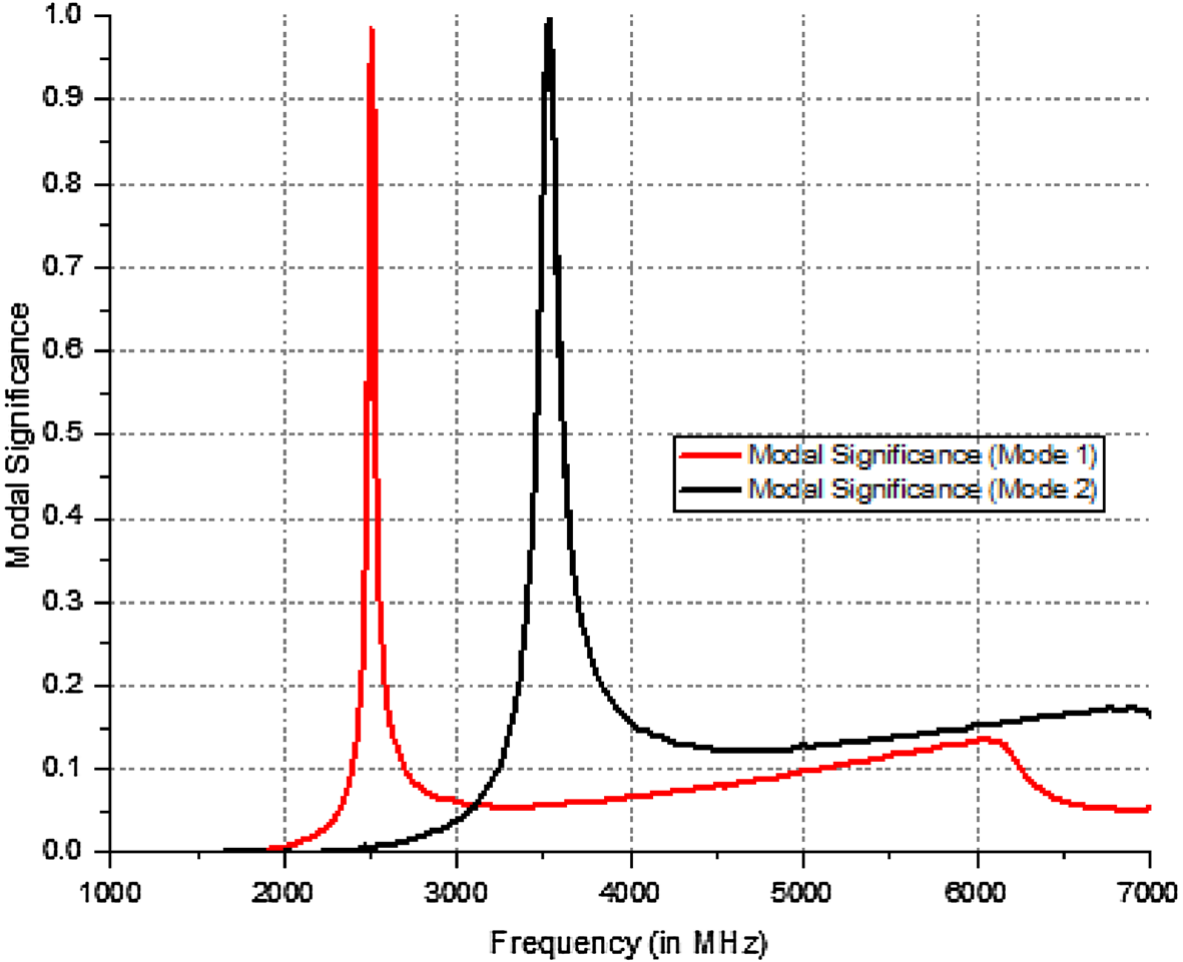
Extended elliptical patch modal significance (radius = 11.50 mm).

Dual mode Dual polarization is produced using mode separation from a circular patch to an extended elliptical patch. The circular patch exhibits two prominent modes that resonate at 2.5 GHz. However, when the patch is transformed into an extended elliptical shape, these modes emerges at 2.5 GHz and 3.5 GHz. This is illustrated with the modal significance plot in Fig. 2. The coaxial feed achieves optimal impedance matching compared to microstrip feed. At 2.5 GHz and 3.5 GHz, the antenna delivers horizontal and vertical polarization, respectively. This research compares the experimental results with the simulated results regarding the S parameters, gain, and efficiency of the suggested prototype.

To accomplish dual-band operation, a pair of small circular patches is positioned along the horizontal axis of the main circular patch. This configuration leads to the formation of a higher-order resonating mode. The modification of the circular patch radius leads to horizontal polarization in the depicted antenna. By stimulating a pair of orthogonal modes for the different bands, an antenna with a dual-band is generated. For determining the orthogonal modes of a circular antenna, the cavity model theory is implemented. CMA is the procedure utilized to validate these modes.

### Characteristic mode analysis

**Figure 3:**
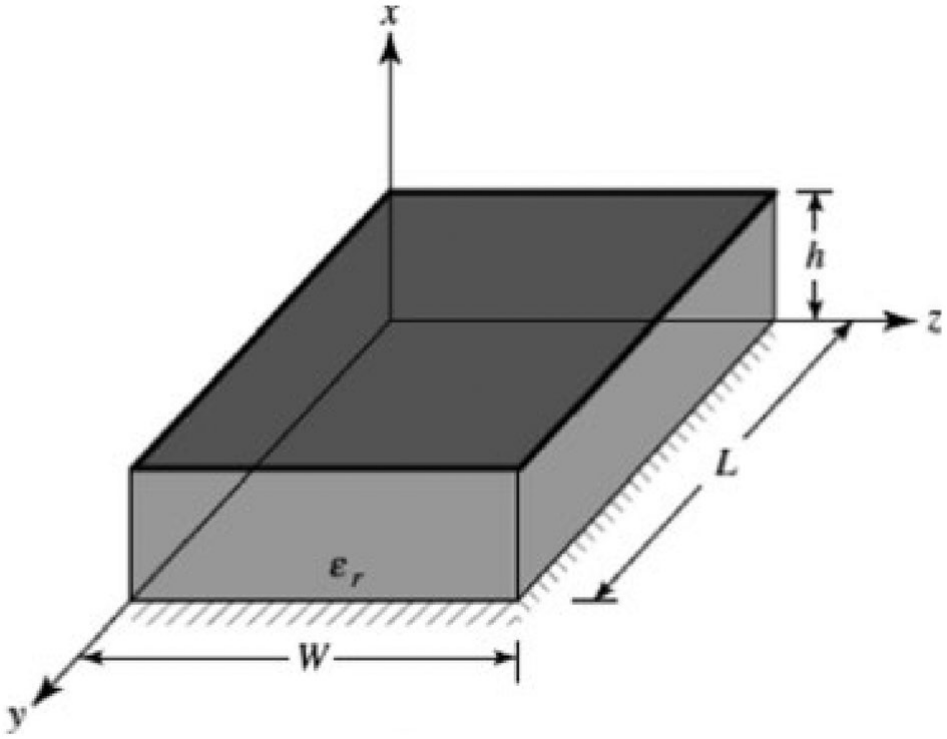
CMA-based cavity model approach to dual-mode antenna design.

CMA is an exceptionally efficient method employed to examine antennas, irrespective of their specific configuration. An essential characteristic of computational electromagnetics is its capacity to offer valuable insights into the operations of antennas via the evaluation of their eigenvalues. The acquired eigenvalues (λ), are a critical component of CMA. The eigenvalues are important in enabling the analysis and assessment of the antenna's radiation properties across multiple modes. CMA's objective is to provide comprehensive analysis and interpretation of the complex electromagnetic characteristics displayed by antennas. The micro strip patch antenna resembles a dielectric cavity and displays a higher-order resonance mode when this technique is applied. A more precise computation within the dielectric substrate, and the normalized fields is feasible by utilizing the electric and magnetic boundaries illustrated in Fig. 3. The resonance of the cavity is denoted by the formula.

1 $${\left({f}_{r}\right)}_{mnp}= \frac{1}{2\pi \sqrt{\mu \epsilon }} \sqrt{{\left(\frac{mn}{h}\right)}^{2}+{\left(\frac{n\pi }{L}\right)}^{2}+{\left(\frac{p\pi }{w}\right)}^{2}}$$

The number of half-cycle field variations in the x, y, and z directions is denoted by the variables m, n, and p, respectively.

The number of actual current modes for any orthogonal geometry is determined by characteristic mode analysis. The materials should be lossless, and this analysis is not influenced by the feeding technique. Modes of characteristics are computed numerically utilizing surface integration equations and the Method of Moments (MoM). Modal significance and characteristic angle are two crucial parameters provided by CMA that facilitate design.

Eigenvalue equation is utilized to determine the characteristic modes.

2 $$\left[\text{X}\right] \overrightarrow{{J}_{n}}={\lambda }_{n} [\text{R}] \overrightarrow{{J}_{n}}$$

In the context of the conducting substance's Z-matrix, the parameters R and X denote the real and imaginary components. The current characteristics of the conducting body are determined by

3 $$\overrightarrow{J}={\Sigma }_{n=1 }^{N}{\alpha }_{n}\overrightarrow{J}$$

$${\alpha }_{n}= \frac{<{J}_{n}{E}^{i}>}{<1+j{\lambda }_{n}>} = \frac{{v}_{n}}{1+j{\lambda }_{n}}$$

The n-th mode’s modal significance can be determined by consulting ^1^.

4 $$\text{MS}=\left|\frac{1}{1+j{\lambda }_{n}}\right|$$

**Figure 4:**
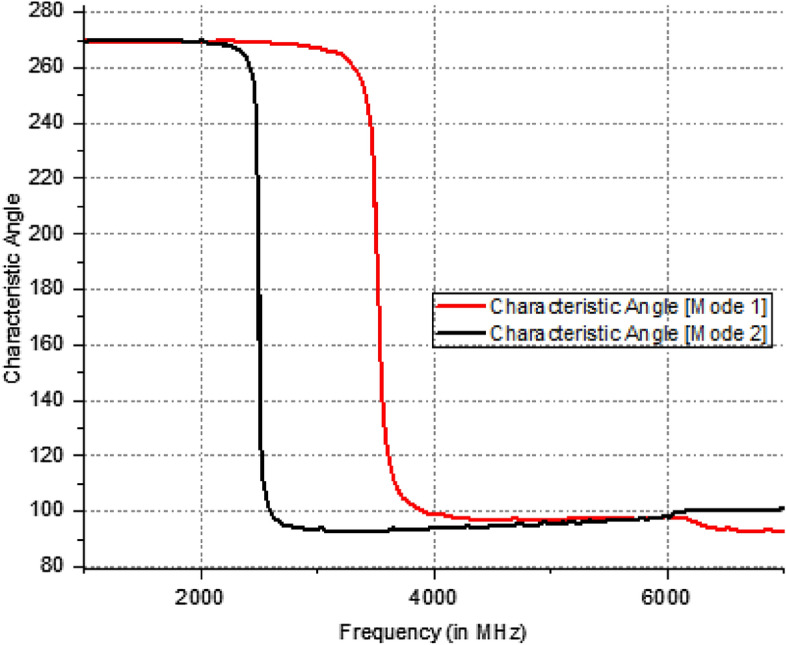
Characteristic angle of the extended elliptical patch.

**Figure 5:**
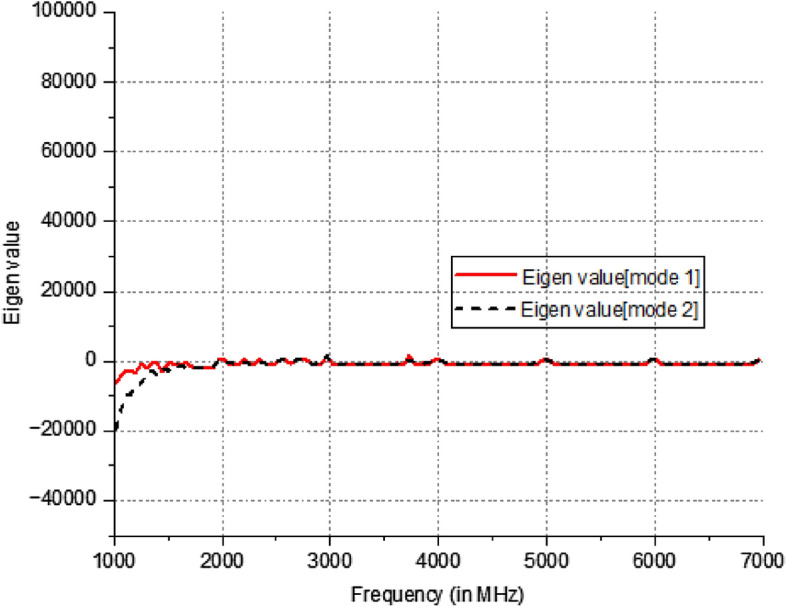
Eigen value of the extended elliptical patch.

**Figure 6:**
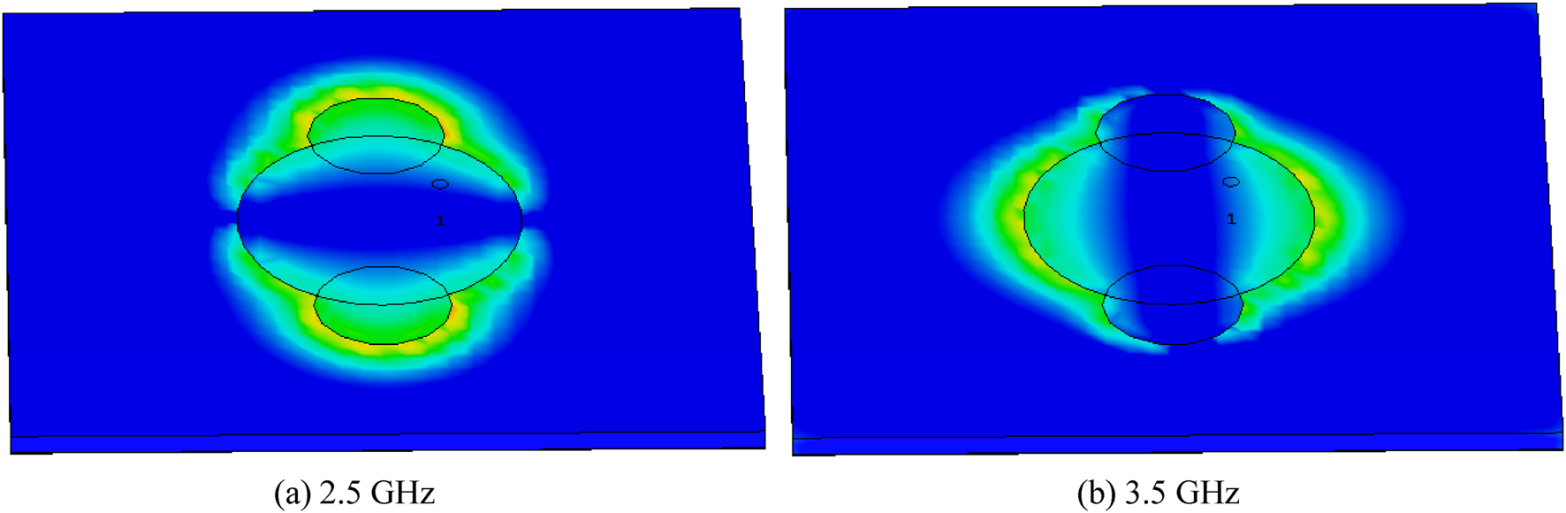
Current distribution over the antenna for both modes.

The Eigen value of the modes is denoted by λ_n_. When MS is greater than $1/\surd 2$, radiation occurs. The method by which the characteristic angle is acquired is Eq. (5). At the operating frequency, the characteristic angle of the radiating mode is 180°. Figure 4 illustrates the characteristic angles of models 1 and 2, revealing that the corresponding radiation frequencies for these models are 2.5 GHz and 3.5 GHz. Figure 5 proposes a eigen value of the extended elliptical patch. Furthermore, Fig. 6 illustrates the current intensity (distribution of electric field) for the designed antenna in relation to models 1 and 2.

5 $$\alpha = {180}^{0}-{\text{tan}}^{-1}({\lambda }_{n})$$

In consideration of expanding the bandwidth of the proposed antenna, the subsequent design equation must be applied

6 $$BW(\% ) = \left( {2.86\left( {\frac{W}{L}.\frac{h}{\lambda }} \right)\left( {\frac{{\varepsilon_{r} - 1}}{{\varepsilon_{r}^{2} }}} \right)} \right)*100$$

**Figure 7:**
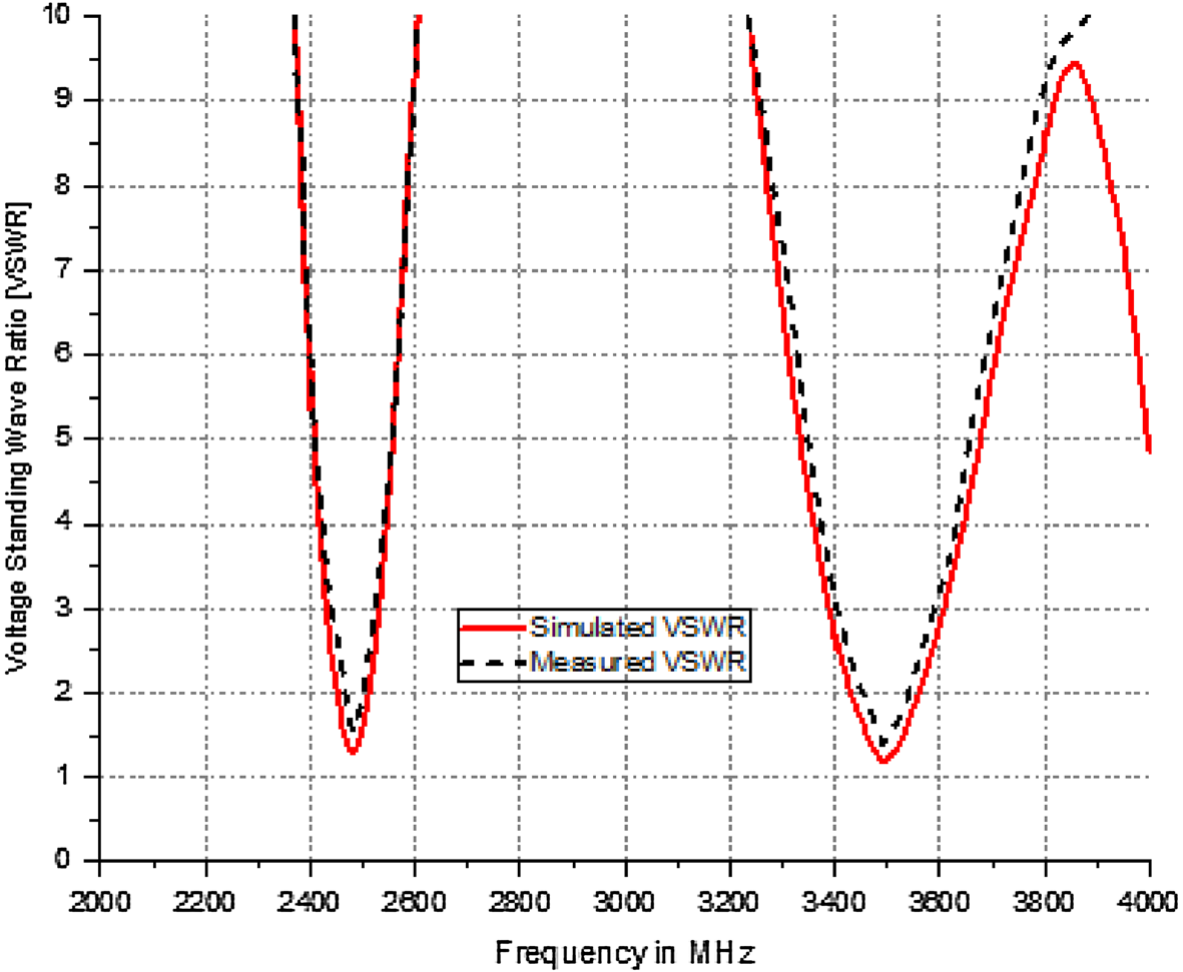
VSWR of the Dual mode antenna (**a**) 2.5 GHz and (**b**) 3.5 GHz.

**Figure 8:**
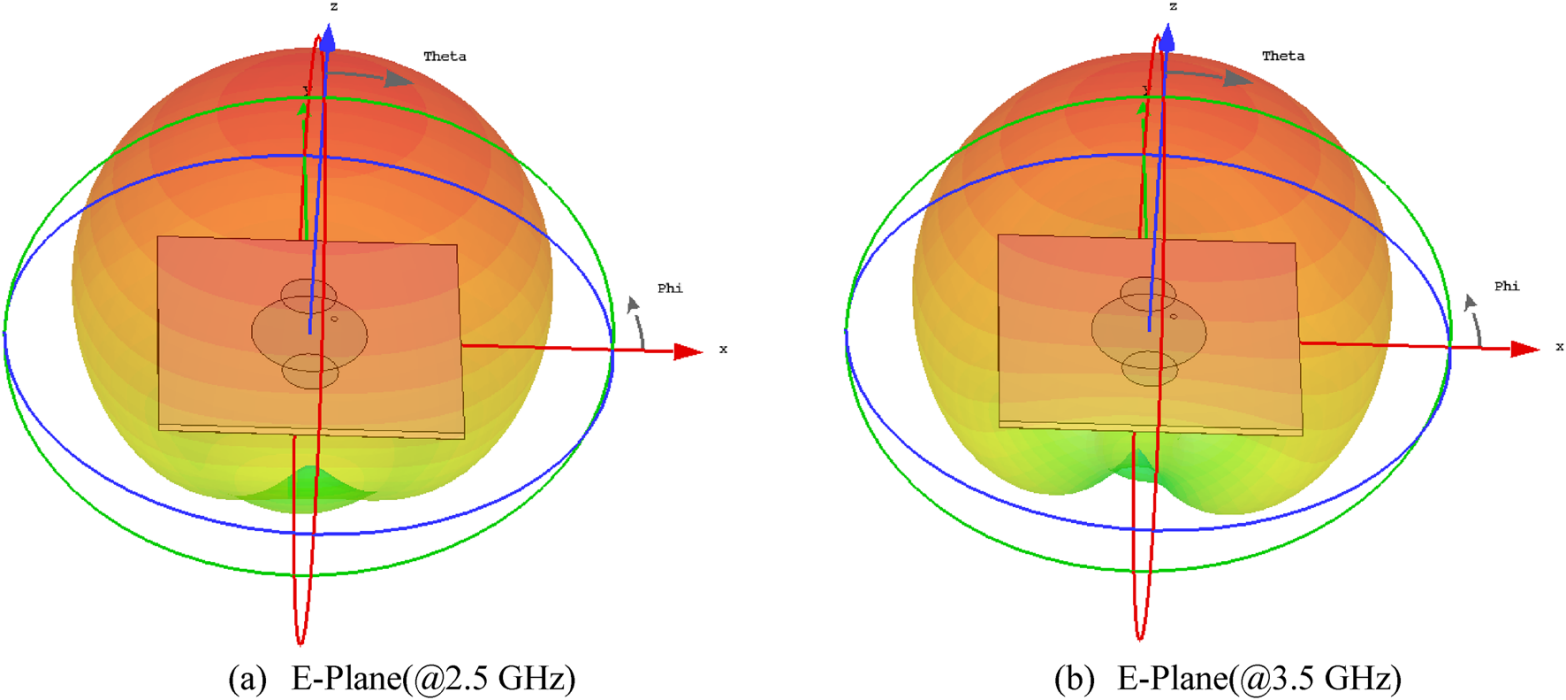
3-D Radiation pattern (Far Filed).

The resonant frequencies vary independently in response to modifications made on the circular patch. As illustrated in Fig. 7, the VSWR falls within the range of 1–2 for the frequency range of 1–4 GHz. The 3D radiation pattern identified at 2.5 GHz with horizontal polarization and at 3.5 GHz with vertical polarizations is illustrated in Fig. 8.

In accordance with above picture, the maximal gain at 2.5 GHz is 4.72 dBi, while the radiation at 3.5 GHz exhibits a directional pattern with gain of 5.31 dBi. To obtain radiation patterns, an azimutal and elevation patterns are utilized.

### Results and discussions

**Figure 9:**
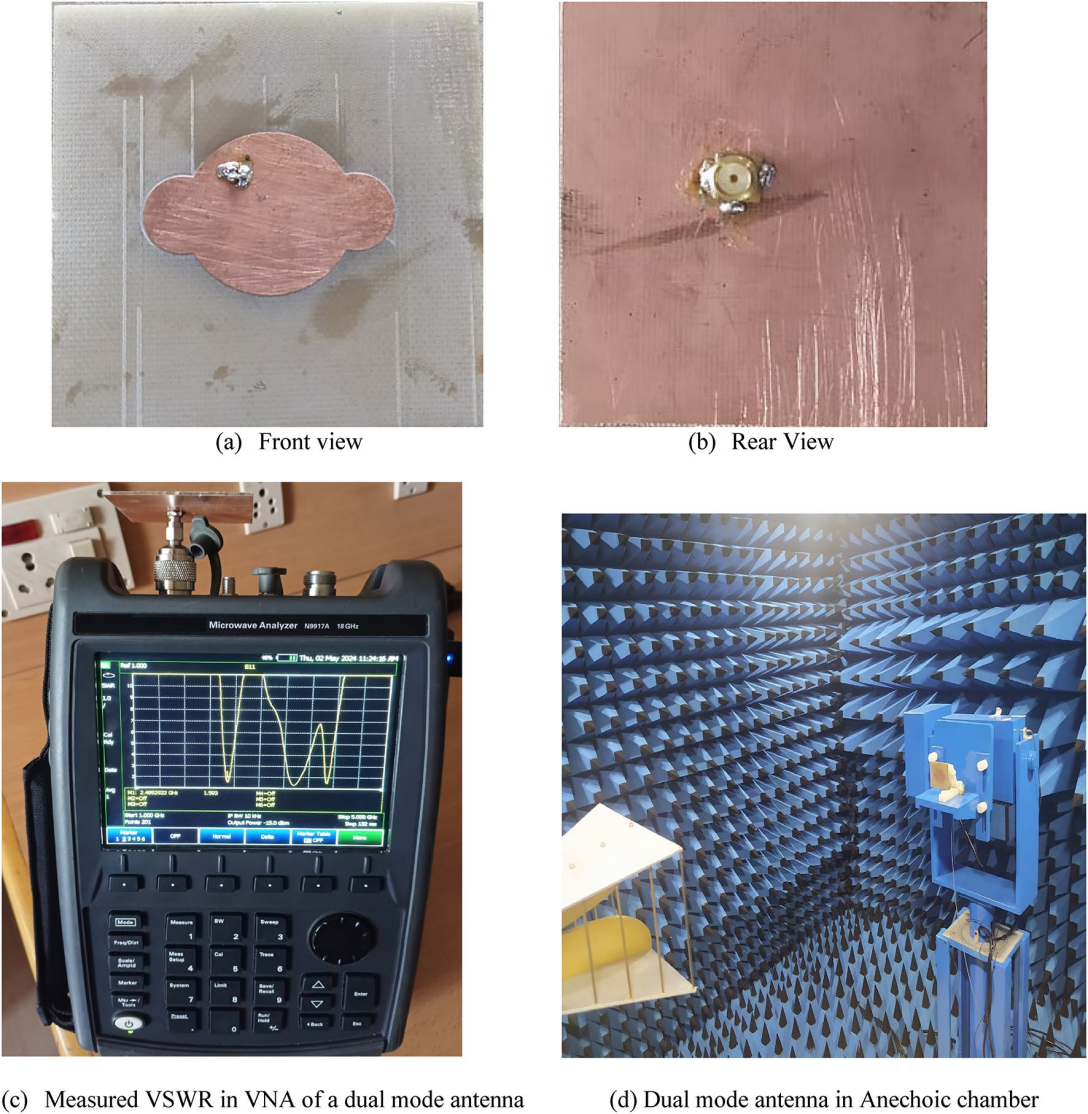
Fabricated view of dual mode antenna.

**Figure 10:**
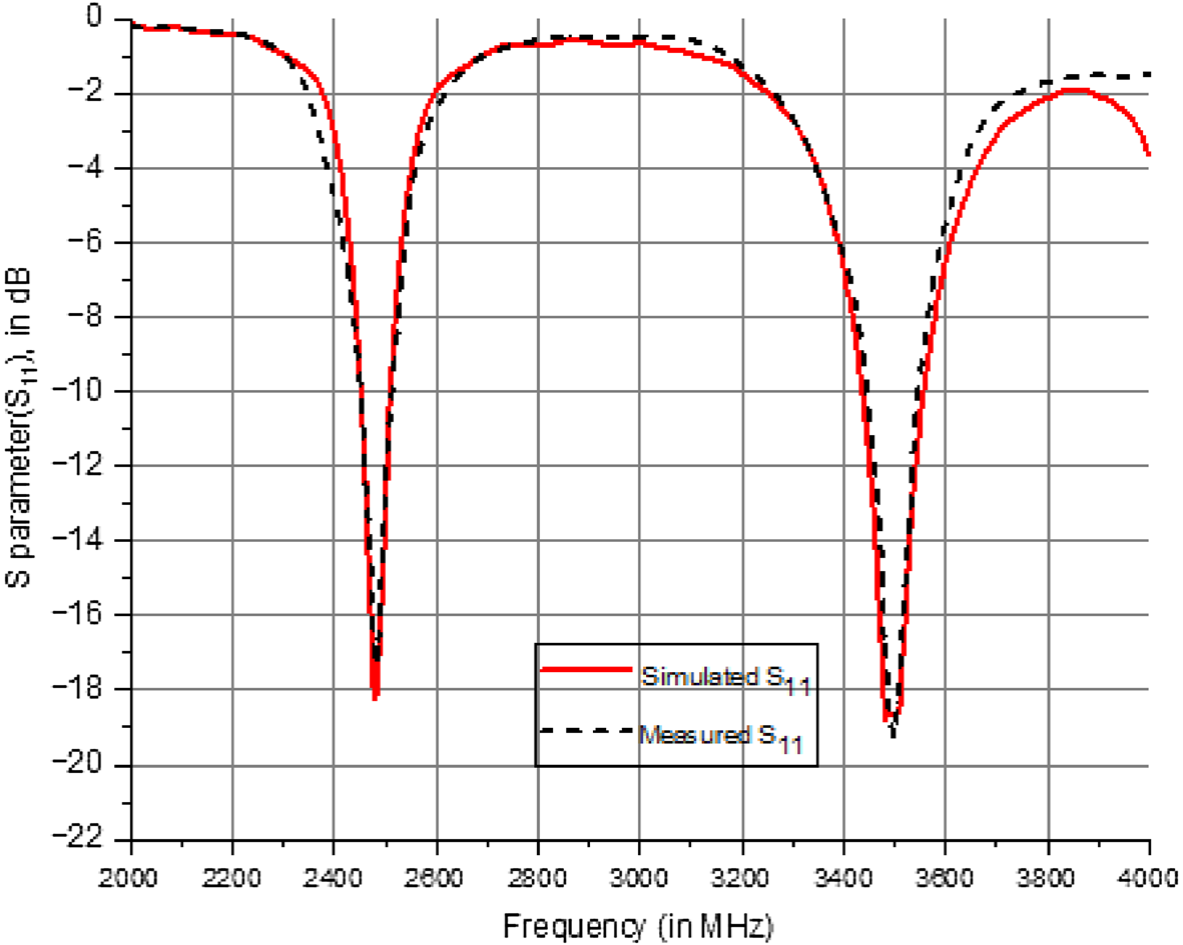
Comparative analysis of measured vs simulated S-parameter (S_11_ in dB) characteristics.

As illustrated in Fig. 9, the suggested antenna is fabricated and evaluated. The S11 characteristics of the prototype are illustrated in Fig. 10. As the initial resonance is produced by the circular patch, two orthogonal modes are produced. The higher-order resonating frequency can be observed through the mode separation of the extended elliptical structure. Lower order resonance is produced by circular patch antenna with horizontal polarization and higher order resonance is produced with vertical polarization. Both simulated and measured values for both resonant modes are identical in the outcome. The − 10 dB bandwidth of 110 MHz (2420–2530 MHz) is centered at 2.5 GHz and 170 MHz (3410–3550 MHz) is centered at the 3.5 GHz frequency range.

**Figure 11:**
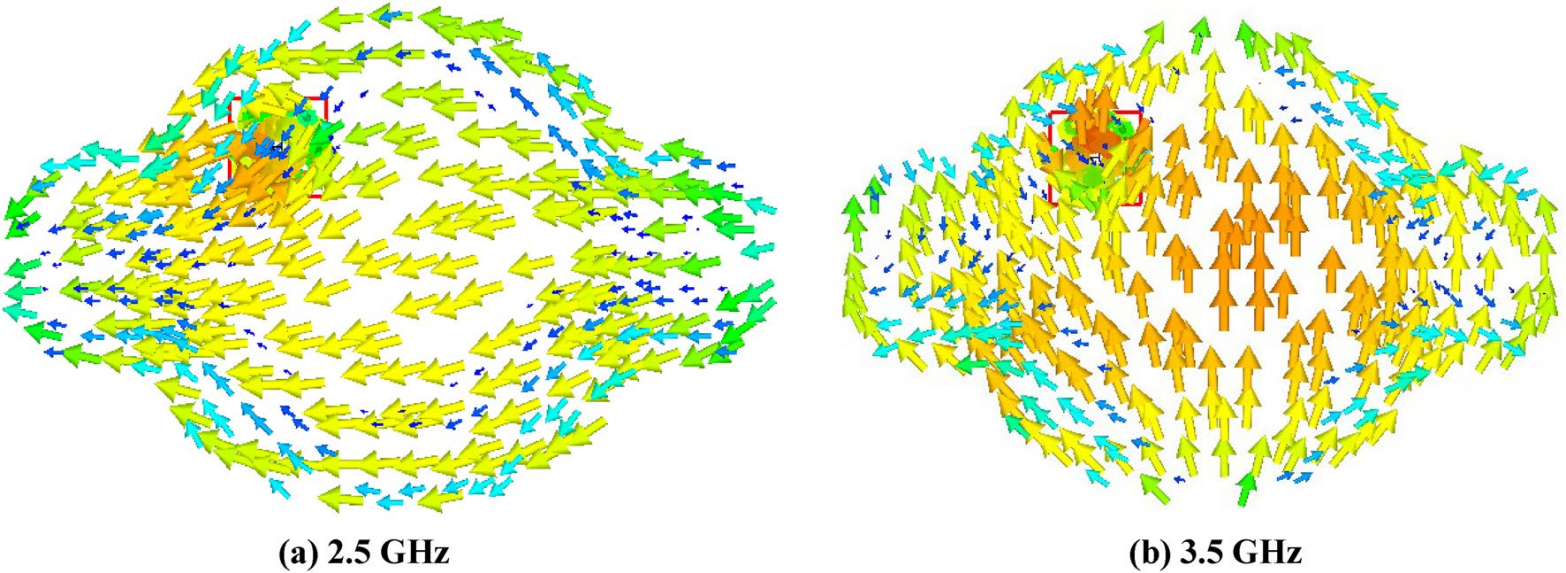
E-field of the antenna at various frequencies.

The antenna's current distribution is illustrated in Fig. 11. The graph demonstrates that the current density is greater at 3.5 GHz, indicated by the red color in the inner region. A similar pattern is observed in the outer patch at 2.5 GHz. A coaxial line is connected to the back side of the antenna using a SMA RF connection, which is soldered in place. The experiment reveals that appended circles result in a reduction in the resonance frequency. When the radius of the circles is altered, the resonant frequencies undergo independent changes.

**Figure 12:**
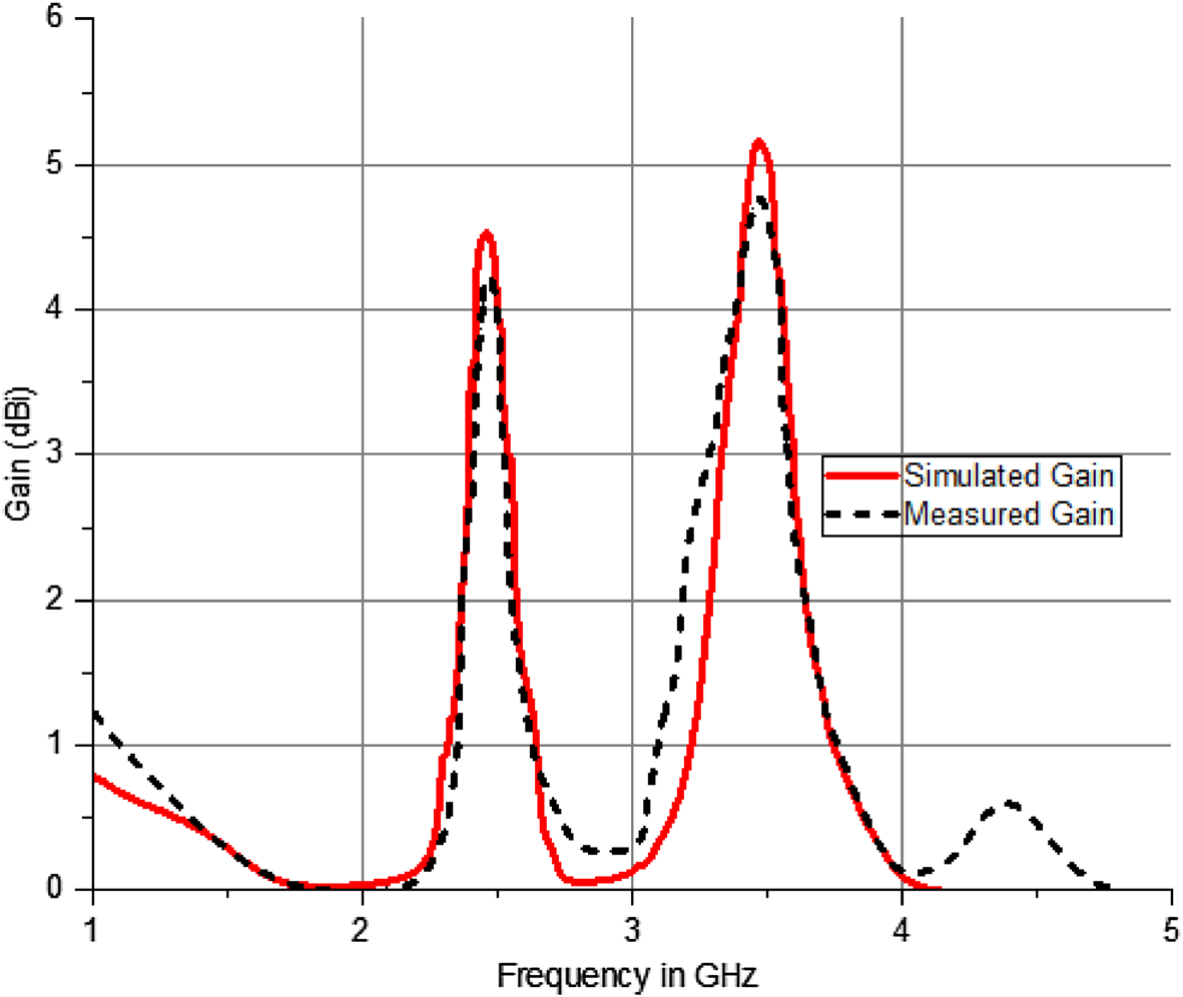
Comparative Analysis of Gain of the dual mode, dual polarized antenna.

The maximum gain of the antenna is 5.31 dBi when operating at a frequency of 3.5 GHz. The radiation pattern resembles that of a directional. As illustrated in Fig. 12, the peak gain at a frequency of 2.5 GHz is 4.72 dBi.

**Figure 13:**
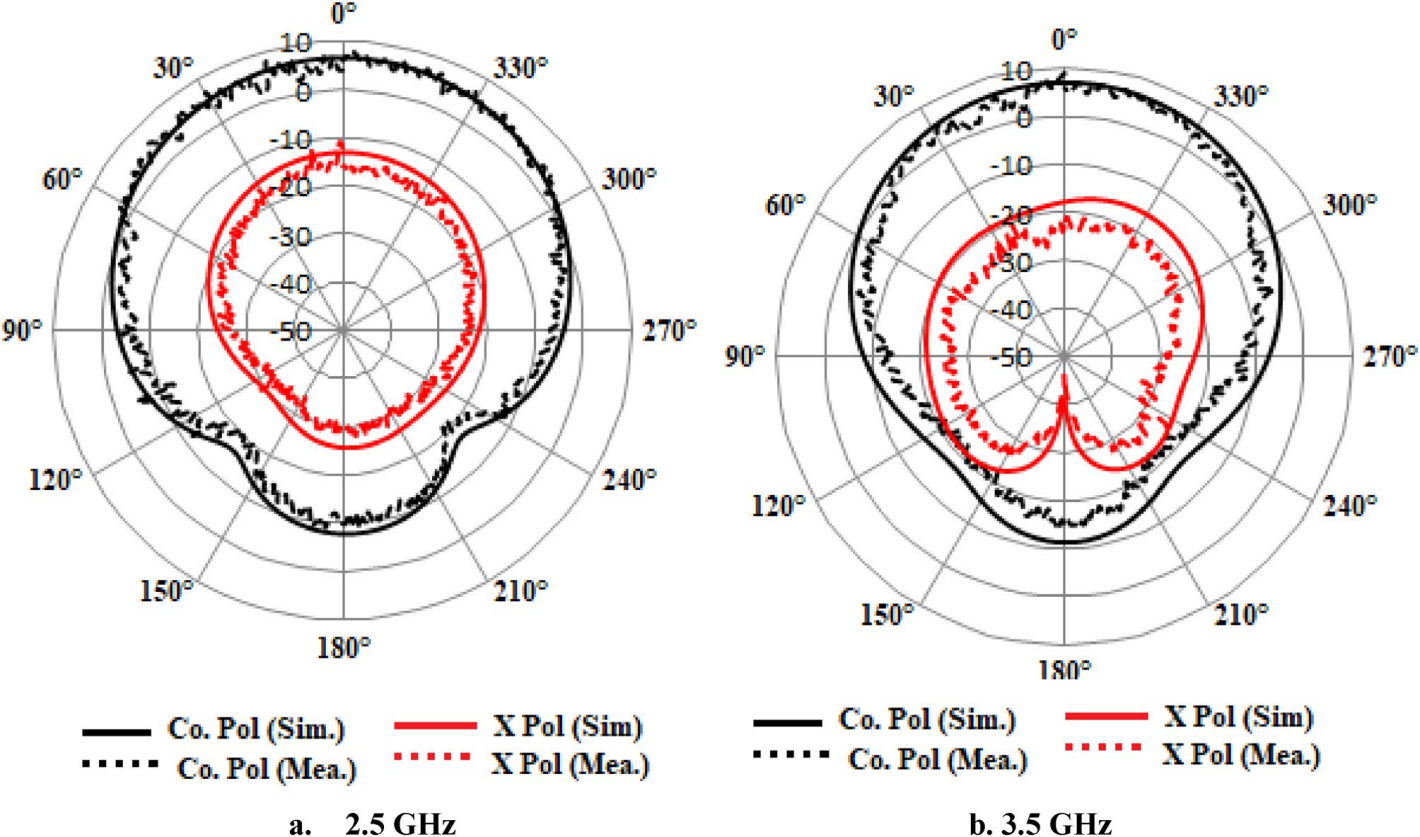
2D radiation pattern of the extended elliptical antenna.

The Finite Integration Technique (FIT) efficiently produces the appropriate radiating modes by utilizing a single coaxial feed line. The suggested antenna resonating at 2.5 MHz for IRNSS and 3504 MHz (S-band) for 5G applications, as determined by measurements. The impedance bandwidths observed are 110 MHz (2420–2530 MHz) and 170 MHz (3410–3550 MHz). The results demonstrate a high level of concordance in terms of reflection coefficient, and pattern and it demonstrates an adequate degree of antenna gain. The CMA analysis is highly effective in identifying resonant modes using a systematic approach. This antenna is a viable option for implementing frequencies below 6 GHz. The suggested antenna exhibits exceptional performance throughout the intended frequency range, including an appropriate impedance bandwidth and a low mutual coupling property. Figure 13 demonstrates that the cross and co-polarization characteristics are employed to assess the far-field radiation performance. It exhibits exceptional performance, especially in the broadside direction with a large gain. Variations in measurements from simulation findings might occur due to errors in fabrication, feeding, and testing operations.

**Figure 14:**
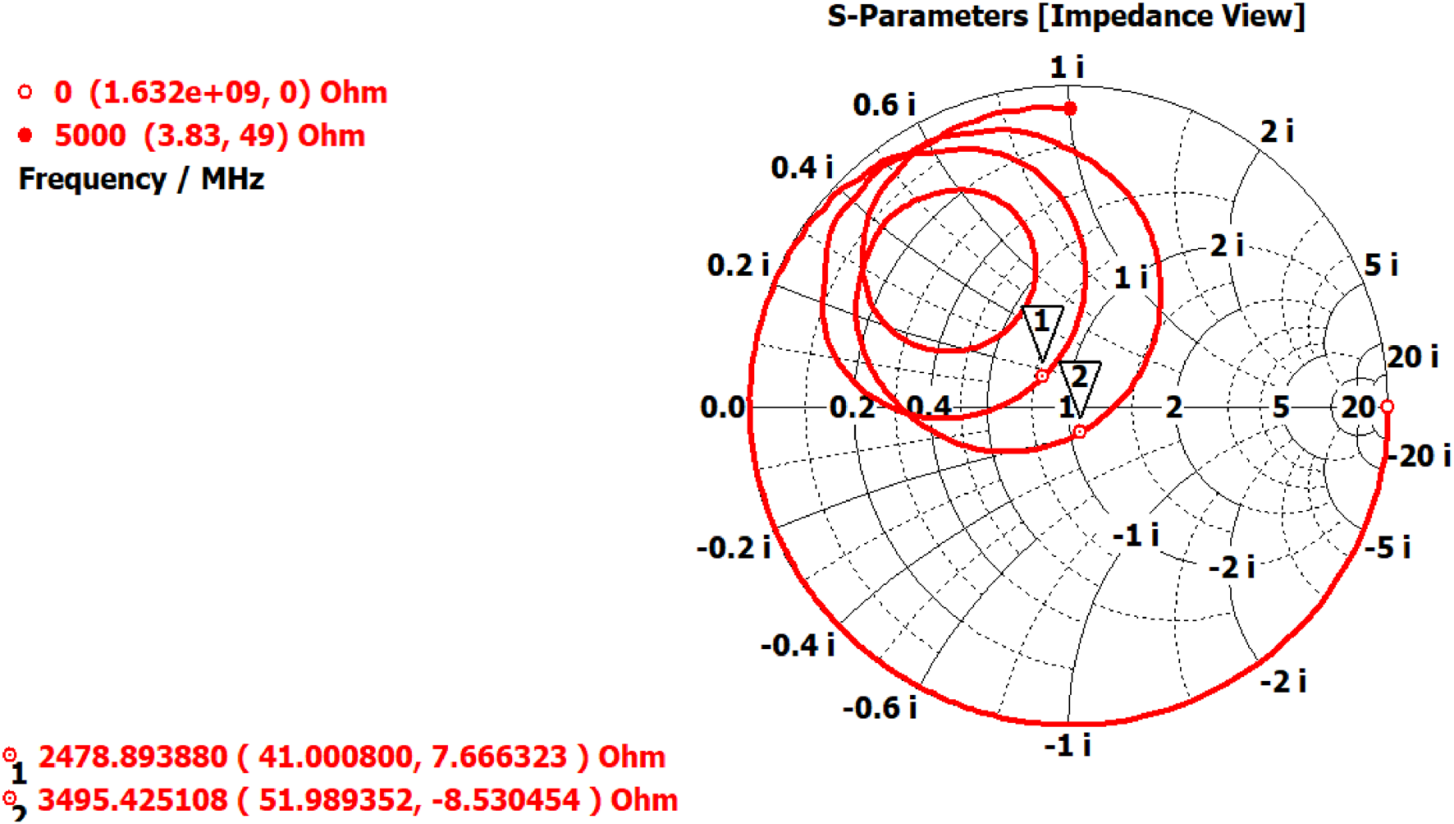
Impedance matching characteristics of proposed antenna (Extended elliptical shape).

Figure 14 illustrates that the Smith chart of the proposed antenna indicates a successful match between the transmission line and the antenna's input impedance. The input impedances of dual-band antennas are (41.6 + j7.3) ohms at 2.5 GHz and (51.9 − j8.5) ohms at 3.5 GHz. Upon examining the input impedance of antennas, it is evident that antennas operating at 2.5 GHz possess a real impedance of 41.6 Ω and an imaginary impedance of 7.3 Ω, indicating an inductive nature. Similarly, an antenna resonating at a frequency of 3.5 GHz has a real impedance of 51.9 Ω and an imaginary impedance of 8.5 Ω, indicating a capacitive nature. For optimal antenna matching, the actual part of the antenna's input impedance must be equivalent to the characteristic impedance of the microstrip input line, which is 50 ohms.

## Broadband dual-polarized antenna geometry

Currently, there is a significant focus on circularly polarized (CP) omnidirectional antennas due to their capacity to efficiently minimize multipath effects, enhance the dependability of wireless networks, and offer extensive signal coverage ^26–28^. As wireless technologies continue to advance, the need for antennas that can operate on several bands is increasing to meet the requirements of various wireless standards. The latest mobile operating system enables unrestricted voice calls and unlimited data transmission through the utilization of 5G technology, which possesses remarkable data processing capabilities ^29,30^. The patch antennas can be conveniently installed by using the single probe feed arrangement, thereby reducing complexity. Reports indicate that achieving dual polarization in single-feed configurations can be accomplished by utilizing miniature electromechanical switches. A design for microstrip patch antenna that utilize multiple PIN diodes and U-slots to achieve polarization and frequency diversity with a single feed are investigated ^31–33^. With its compact size, low weight, uniplanar structure, and broad bandwidth, the monopole antenna is utilized in 5G technology. Recently, there have been proposals for wideband antennas that operate in the sub-6 GHz region. It is studied that the orthogonal field components are responsible for circular polarization (CP). In addition, the impedance bandwidth can be enhanced by removing the edges, making virtual connections, and introducing slits along the borders. Achieving high data rates and optimizing channel capacity are critical objectives in wireless systems ^11,34^. Based on the aforementioned outlook, the requirement for a broadband antenna with circular polarization is inevitable. The suggested notched monopole antenna offers enhanced bandwidth in two frequency bands. To enhance the bandwidth, the design incorporates an L-shaped design at the bottom of the plane with parasitic elements. A recent study has examined the use of wideband circularly polarized patch antennas operating at a frequency of 45 GHz ^35,36^. The 3dB axial ratio (AR) bandwidth is 17.3%, whereas the broadside antenna has a maximum bandwidth of 24.9% for |S11|< − 10 dB. The utilization of a T-shaped microstrip line to power a dual-polarized antenna enables the attainment of a wide frequency range, consistent radiation patterns, and a significant increase in gain. Circular polarized antennas should have a broadside 3 dB axial ratio bandwidth (ARBW) that covers the required frequency ^37–39^. This demonstrates the CP's ability to minimize return losses and maximize gain. The CP antenna is capable of operating with the target bands even if they are only partially covered by the 3 dB ARBW. Reconfigurable antennas fall into the following categories: frequency, polarization, and radiation pattern. Reconfigurable antennas possess the ability to change polarization, which greatly enhances signal reception by effectively reducing the negative effects of multipath fading ^40–42^. Moreover, modern communication systems favor the use of multiband reconfigurable antennas.

Hence, the primary aim of this project is to create a new microstrip antenna that can be adjusted in terms of both polarization and frequency^43,44^. The present study introduces a small, CP antenna with an axial ratio bandwidth (ARBW) of 3 dB. This antenna is capable of supporting both 5G and mid-band communication standards. The V-shaped slot is introduced at the bottom of the antenna to facilitate the injection of currents with equal amplitudes and a 90° phase difference into the radiating patch at particular locations. This adjustment is made to generate circular polarization at the desired frequency bands with maximum gain. The results, both experimental and theoretical, suggest that it is possible to attain gain values higher than 5.3 dBi within the operational range for both standards in the context of 5G mid-band applications.

**Figure 15:**
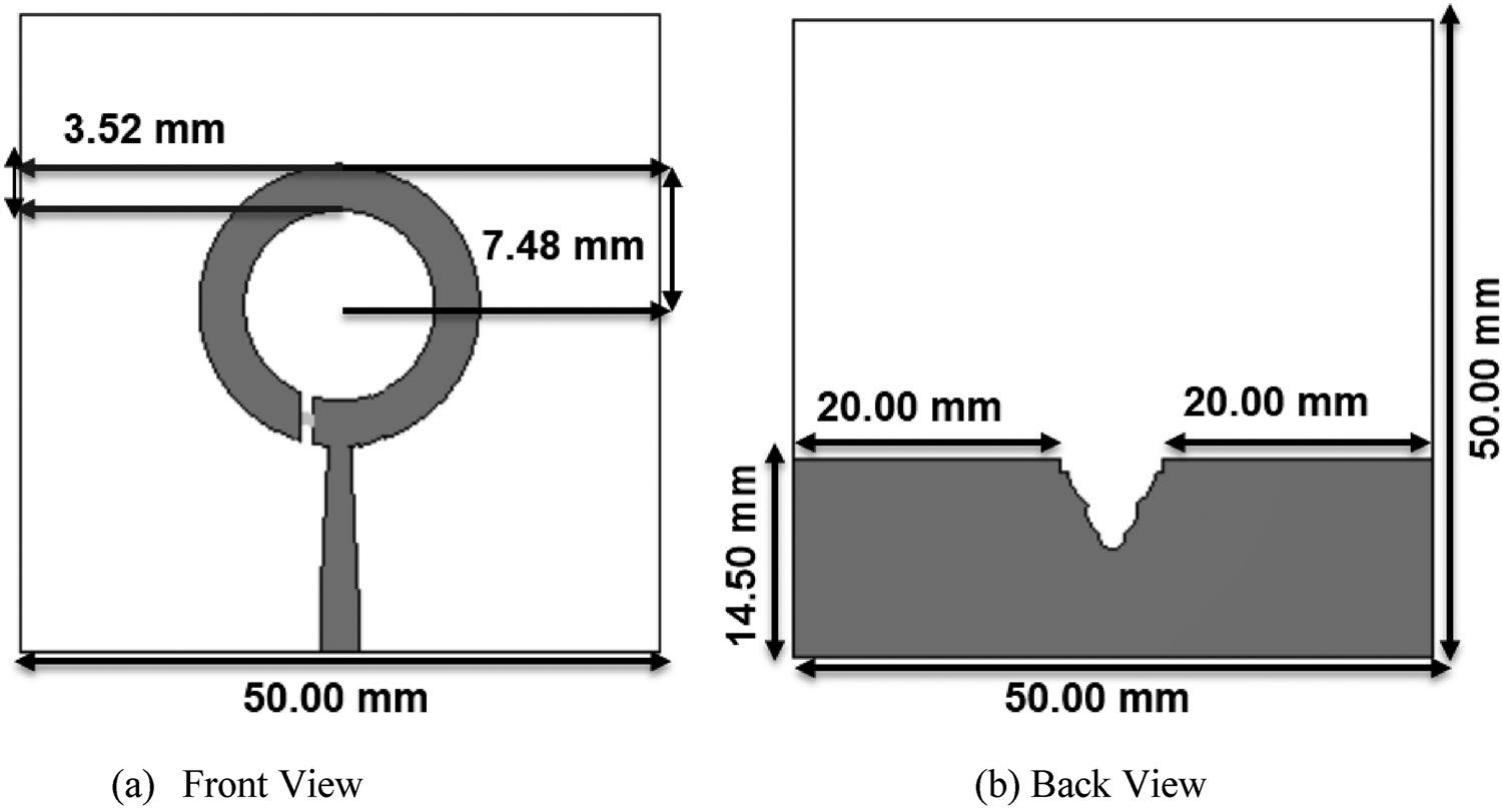
Physical geometry of the broadband dual-polarized antenna.

Figure 15 displays the schematic of the antenna, depicting a circular ring with a stepped feed positioned above the radiator. The bottom of the ring features a partial ground. By utilizing slots and modifying the ground plane, impedance matching is accomplished in the broadband range. The antenna is constructed using FR4 material, characterized by a dielectric constant of 4.4 and a thickness of 1.6 mm. The physical footprint is approximately 50 mm × 50 mm.

**Figure 16:**
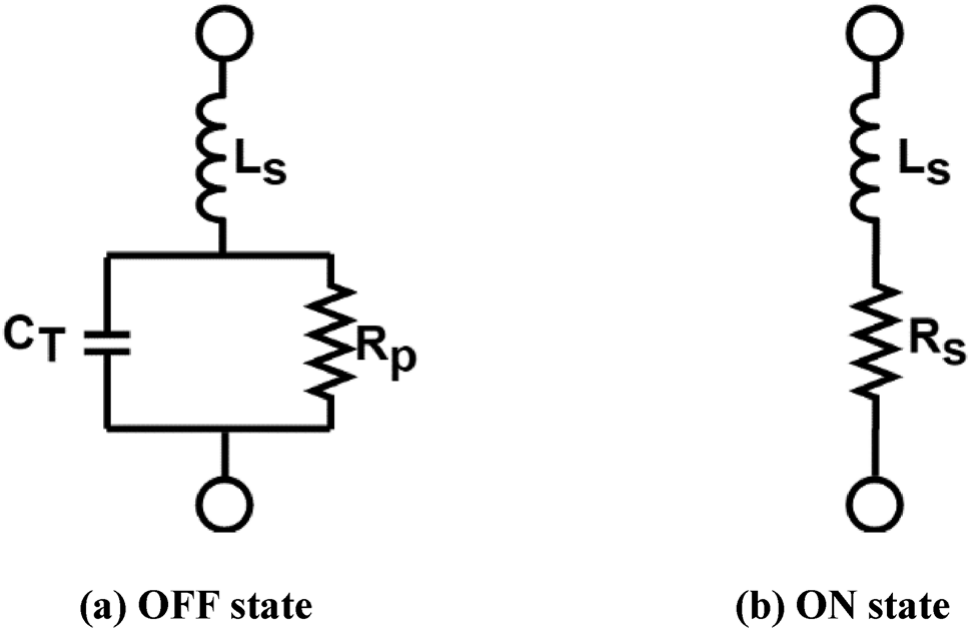
RLC model PIN diode.

**Figure 17:**
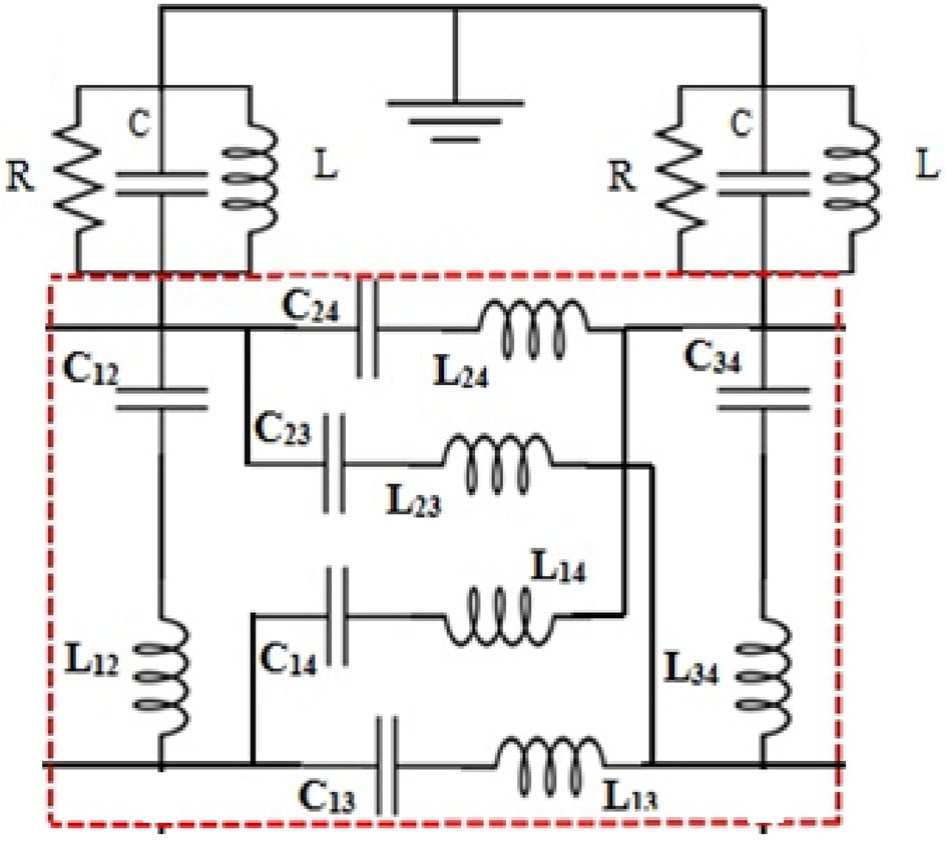
Equivalent circuit of RLC model PIN diode.

**Figure 18:**
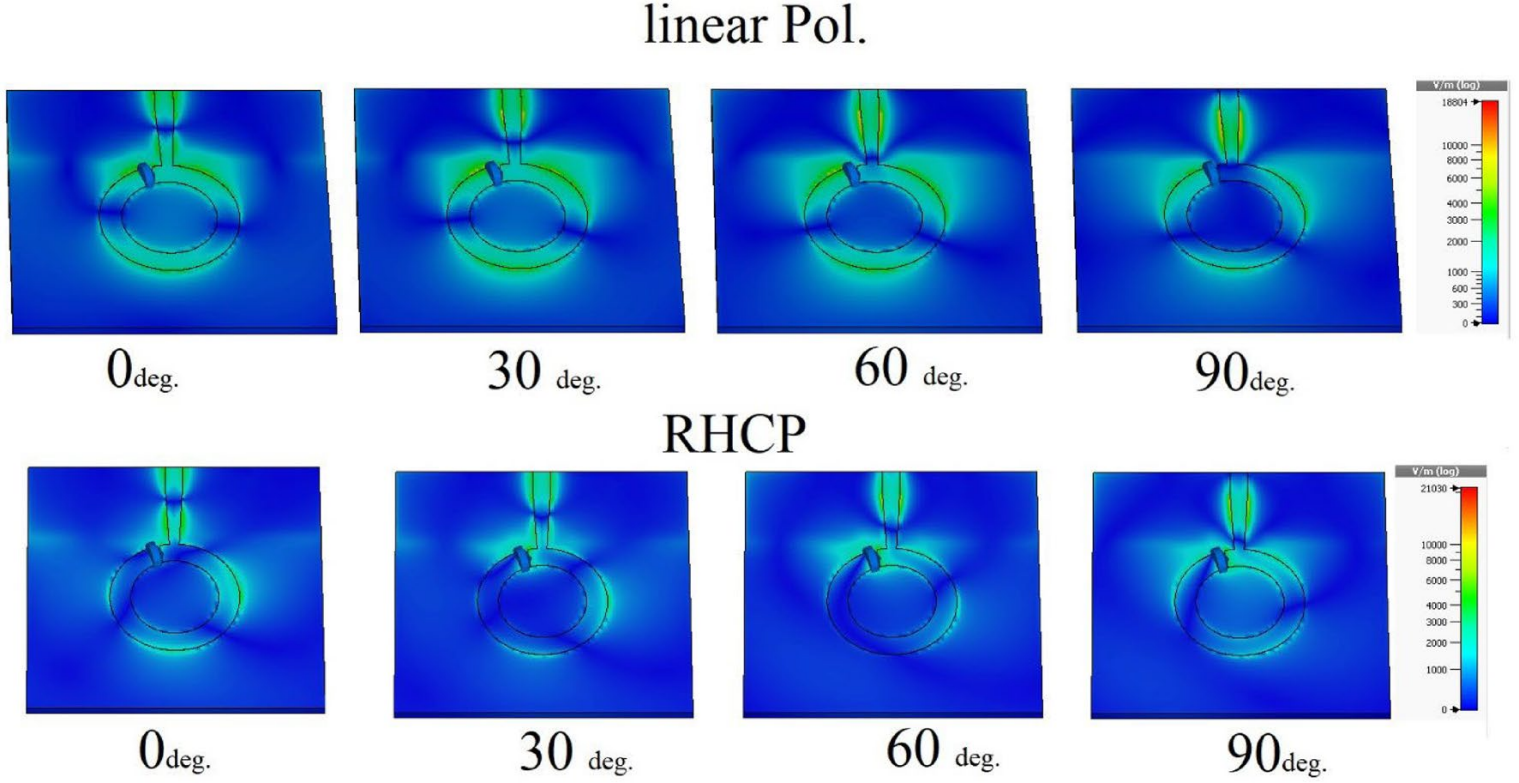
3D E-field distribution of the suggested antenna.

**Figure 19:**
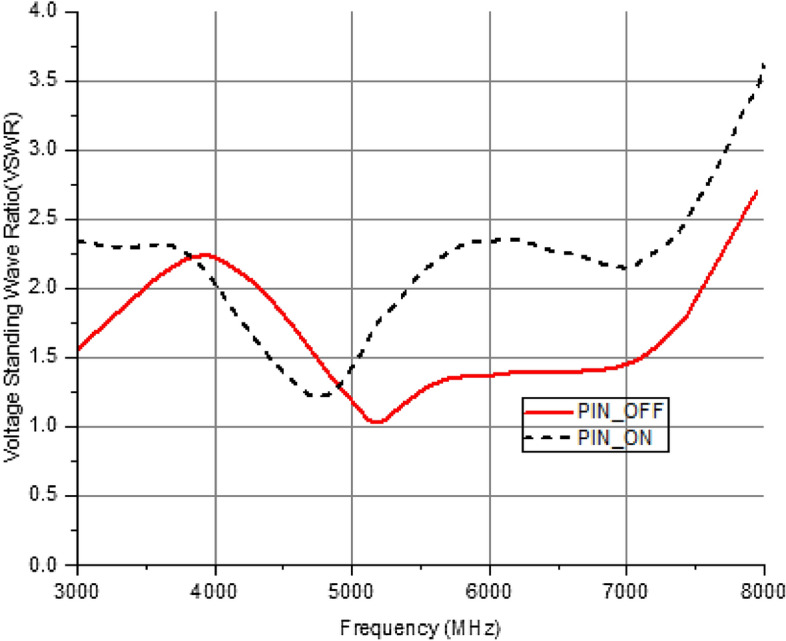
VSWR of an antenna with dual polarization.

The ring-shaped antenna is positioned adjacent to the feed point to accommodate the placement of the PIN diode. The operational status of the diodes’ equivalent circuit, as depicted in Fig. 16, can regulate the functioning of the device. Furthermore, Fig. 17 displays the equivalent circuit of the suggested dual-band polarized broadband antenna, with the diode obtained from the CST simulator. When the diode is in the active state and has the following values: R = 0 Ohm, L = 1 nH, and C = 1 nF. Figure 18 illustrates the changing resonant properties (distribution of electric field) at different angles for linear and right-handed circular polarization. During this condition, the antenna demonstrates resonance spanning the frequency spectrum from 4.3 to 7.5 GHz. with a reference level of − 10 dB. Conversely, in the OFF state, the circuit has the following values C = 1F, L = 6 KH, and R = 6 KΩ. It resonates within the frequency range of 4.2–5.3 GHz. The impedance-matching properties of the proposed antenna are illustrated in Fig. 19 using a VSWR plot. The plot shows a VSWR of 1 at 5.1 GHz when the diode is turned OFF, and a VSWR of 1.2 at 4.7 GHz when the PIN diode is turned ON.

### Dual-polarized antenna

**Figure 20:**
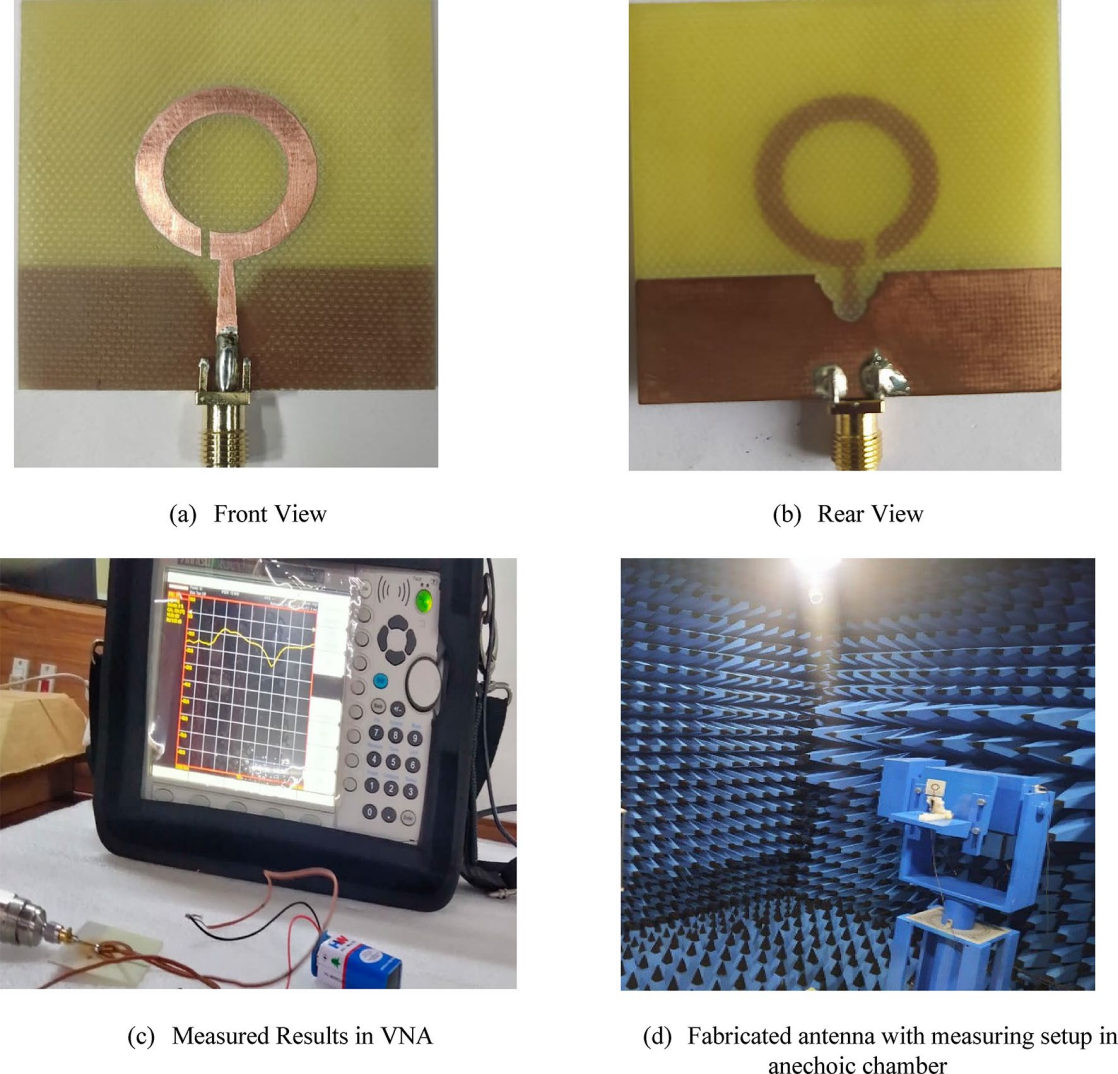
Fabricated antenna with measuring setup in anechoic chamber.

To assess the antenna’s figure of merit, experiments are performed on a prototype of the antenna. Figure 20 depicts the antenna positioned in the anechoic chamber, together with the measurement holder. A PIN diode is linked to the aperture and is powered by a 9V battery. The diode exhibits a bandwidth of 3.2 GHz, specifically ranging from 4.3 to 7.5 GHz when it is in the ON state. Conversely, the antenna offers a bandwidth ranging from 4.2 to 5.3 GHz. The observed results are found to be identical to the results obtained from the simulations conducted using CST.

**Figure 21:**
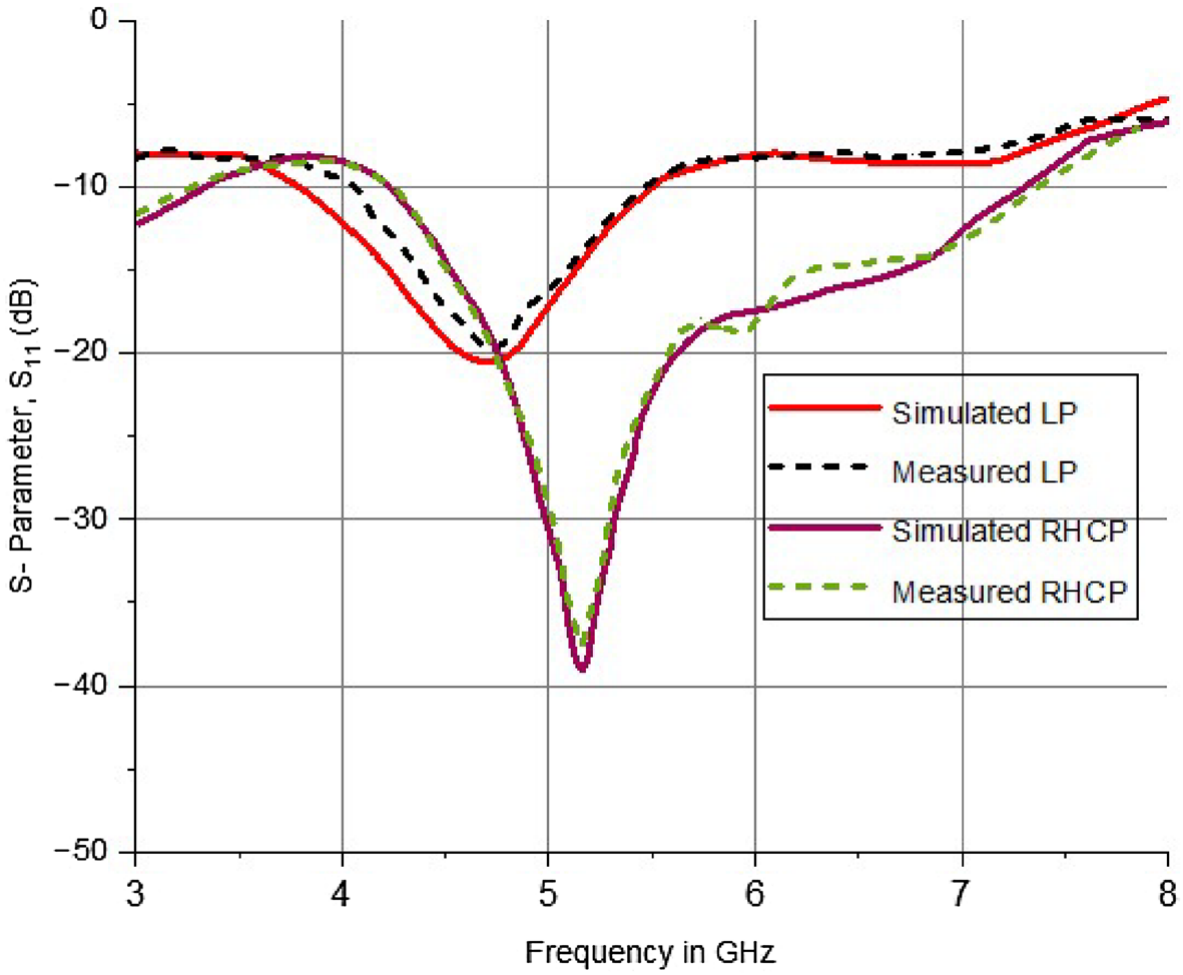
Performance Analysis of antenna showing S-Parameters (S_11_, in dB) for both operating modes (measured and simulated).

The broadband antenna under consideration was fabricated and evaluated within the limits of an anechoic chamber. The comparison was made regarding the performance of the depicted antenna with the reference antenna working in the frequency range of 1–18 GHz and having an impedance of 50 Ω. By implementing entire ground planes, it is feasible to achieve a limited range of frequencies. Conversely, by incorporating V-shaped slots with partial ground planes, it is possible to achieve a broader range of frequencies. The wideband range's impedance matching depends on the influence of V-shaped slots and alterations in the bottom of the plane. When the diode is in the ON state, S_11_ parameters of -39 dB is obtained over a wideband range at a frequency of 5.2 GHz. Conversely, in the OFF state of the diode, a reflection coefficient of − 21 dB is obtained over a narrowband range at a frequency of 4.7 GHz. A vector network analyzer is employed to validate these findings. The performance analysis of antenna showing S-Parameters (S11, in dB) for both operating modes (measured and simulated) is shown in Fig. 21.

**Figure 22:**
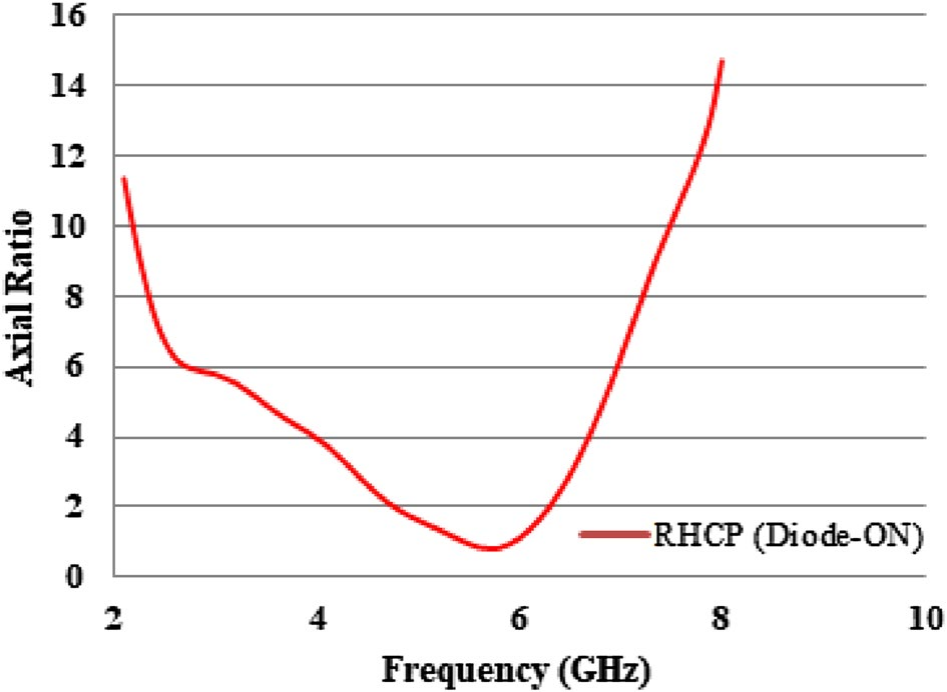
RHCP antenna axial ratio.

In order to operate the antenna, it must be configured in two different ways. The E field distribution at different angles during the active state of the antenna. The obtained E field distributions are displayed at different frequencies and phases to analyze the circular polarization mechanism of the suggested antenna. The maximum currents are recorded in the stepped feed and top side ring of the radiating element. The current vectors on these radiators can yield dominant current vectors in all instances. The resulting current vector undergoes rotation within the range of 0°–90° at various frequencies. Due to anticlockwise rotation in current rotation, the broad side exhibits right-hand circular polarization (RHCP), while the narrow band side exhibits linear polarization (LP). The results were further confirmed by the axial ratio plot depicted in Fig. 22. The specified bandwidth ranges from 4.2 to 6.8 GHz and is measured using a 3 dB reference line. When the diode is not activated, the antenna exhibits linear polarization. The other mode displays an axial ratio that exceeds the 3 dB reference significantly.

**Figure 23:**
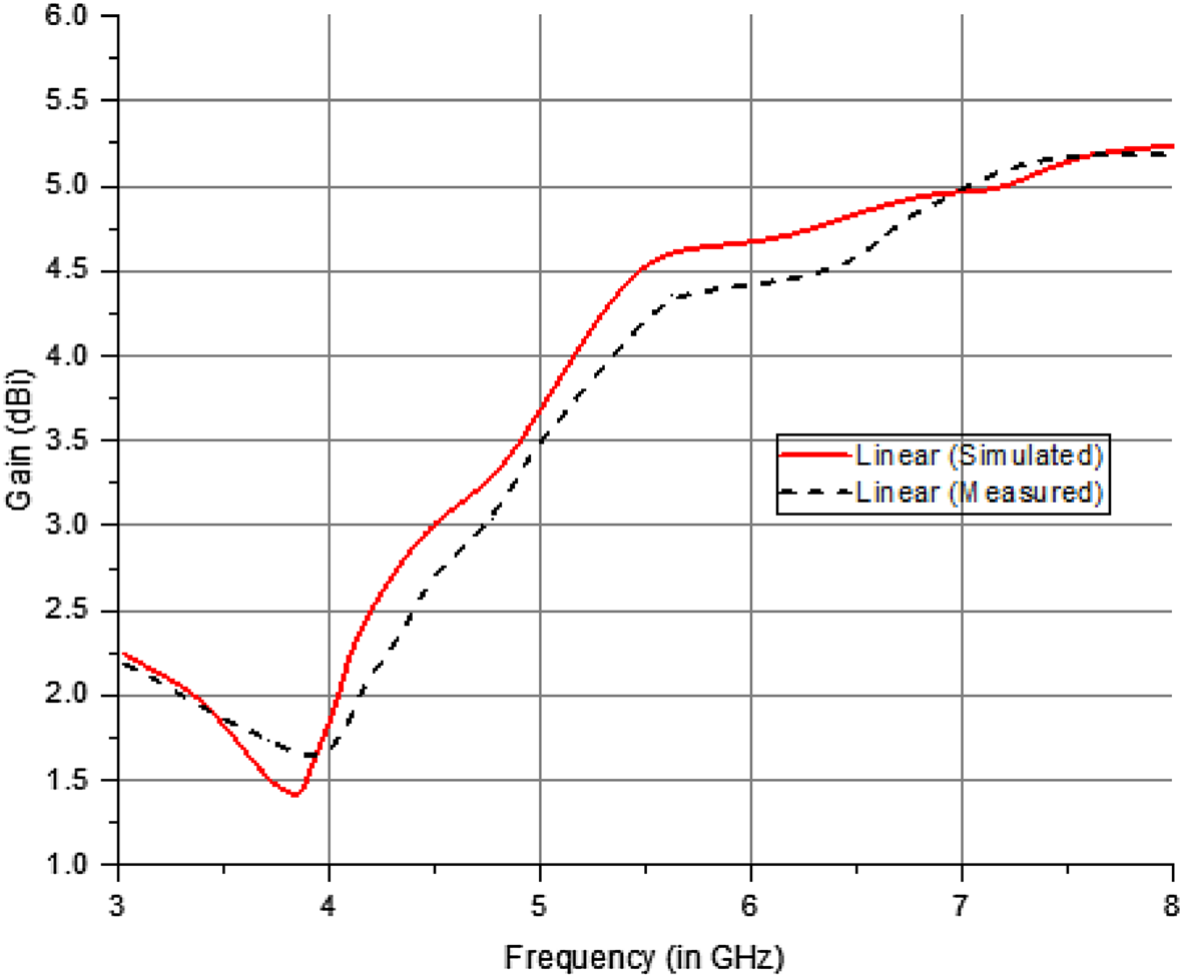
Gain characteristics of suggested antenna (Linear Polarization).

Figure 23 displays the maximum gain (circular polarization) of the recommended antenna under various circumstances of active elements. Typically, the antenna detects signals in the direction where it has the maximum gain level.

**Figure 24:**
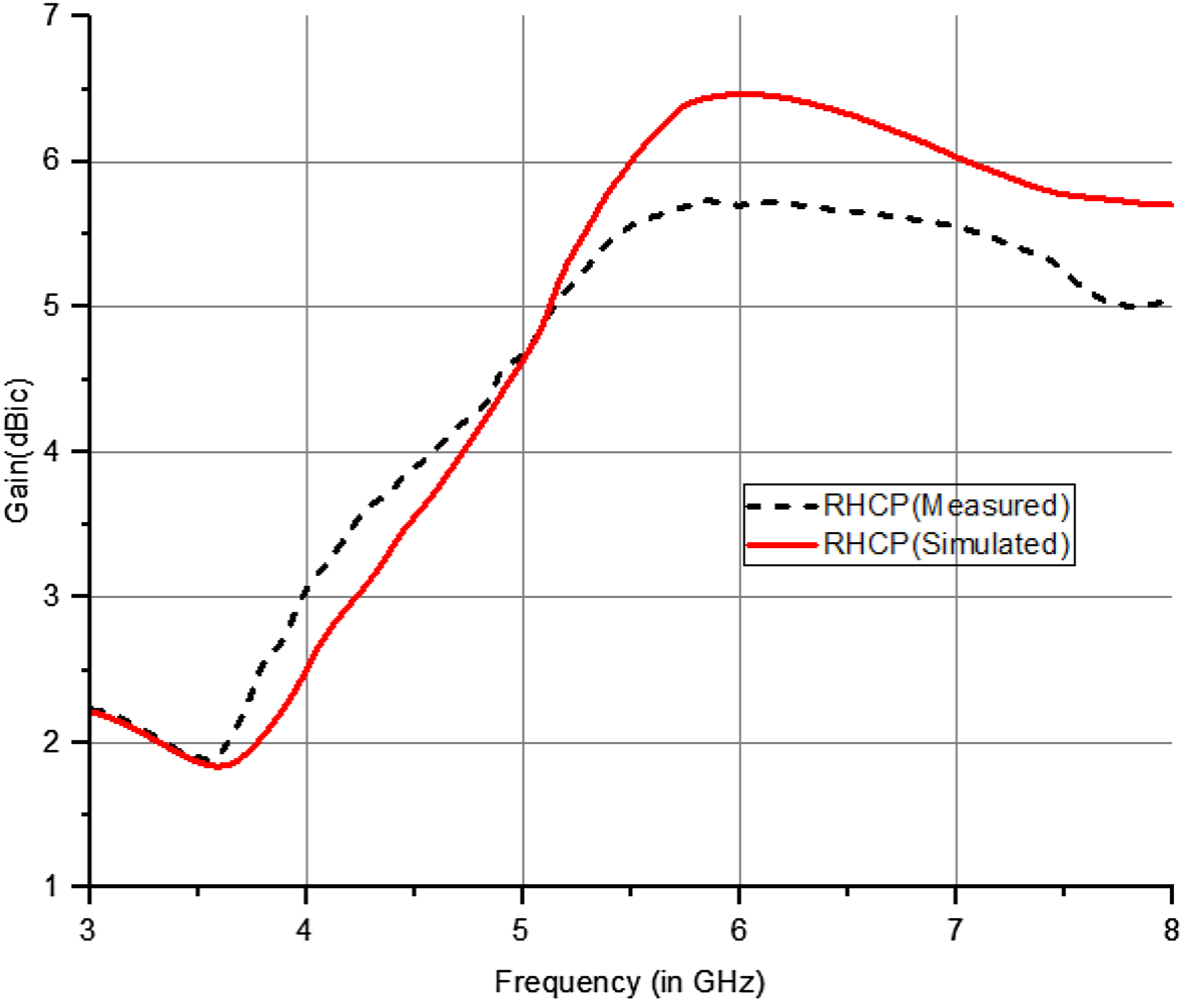
Gain characteristics of suggested antenna (Circular Polarization).

The graph in Fig. 24 clearly shows a substantial increase in the maximum gain at the notched frequencies. Regarding the suggested antenna, it adequately covers the operational bands with an acceptable level of gain. The maximum gain obtained in this case is 6.5 dBic in circular polarization and 5.2 dBi for linear polarization in various operational scenarios of the PIN diode. The experimental and theoretical results indicate that gain values of more than 5.2 dBi are obtained throughout the operational range by 5G mid-band applications.

**Figure 25:**
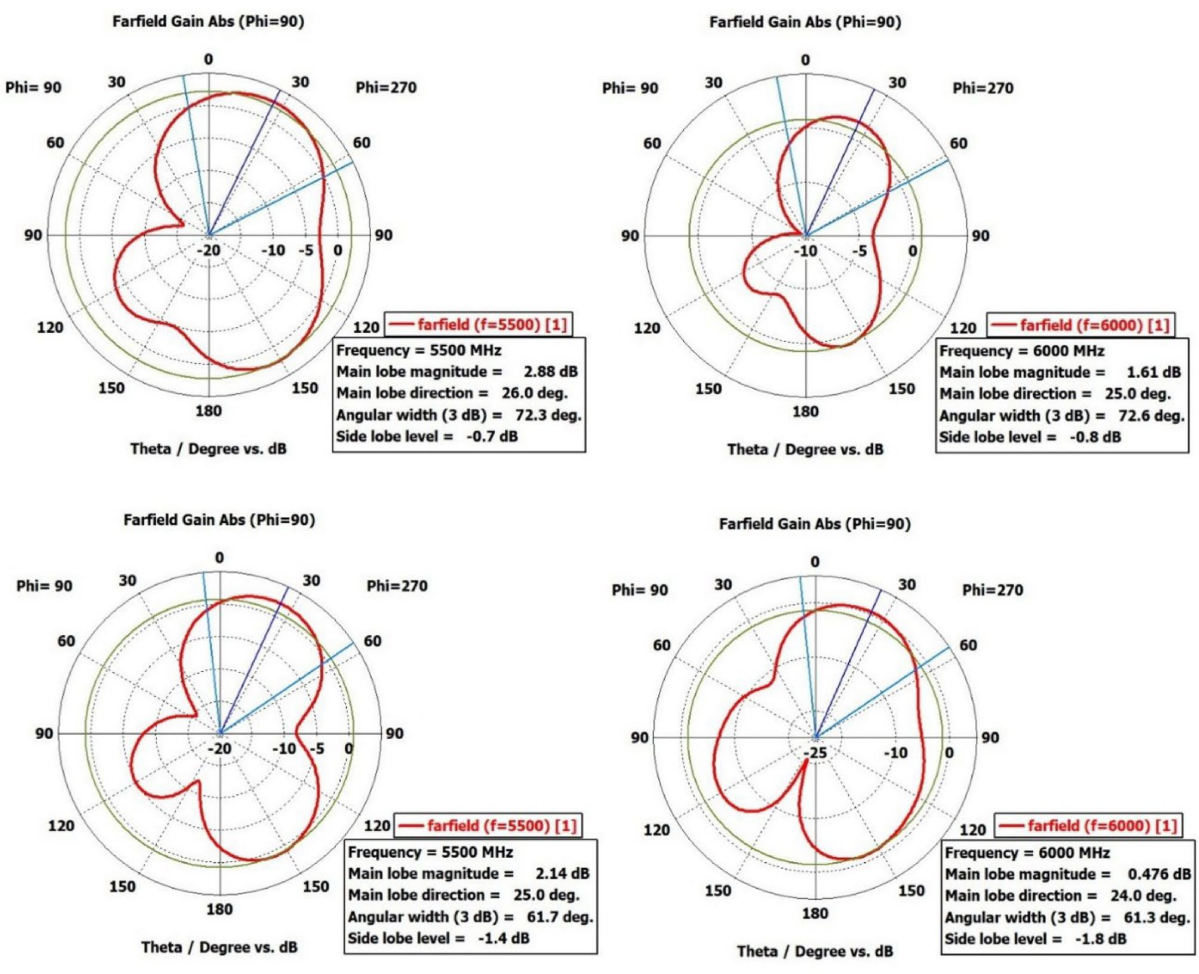
Simulated 2-D Radiation Pattern Dual-Polarized Antenna Geometry (@5.5GHz, @6GHz).

**Figure 26:**
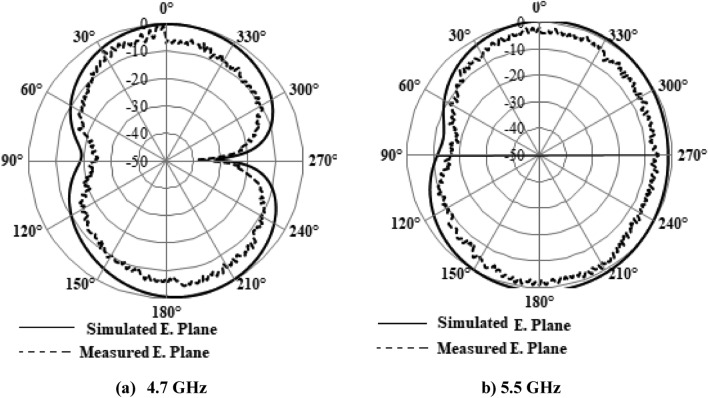
Radiation pattern for 4.7 GHz and 5.5 GHz.

Figures 25 and 26 displays the radiation pattern at the resonating frequencies of 4.7 GHz, 5.5 GHz, and 6 GHz. The axial ratio can be determined by analyzing radiation patterns. The electric field distribution with varying phase angles can be used to confirm either RHCP or LP in the given patterns. Across all angles, the cross-polarization results are lower than the co-polarization results. According to the simulation results, the radiation pattern meets the initial criteria. The designed antenna's overall characteristic parameters are compared with the relevant parameters of existing antennas, and it demonstrates superior qualities, as indicated in Table 1.

**Table 1:** Comparative analysis of suggested antenna with relevant antenna.

Ref No	Resonant Frequency (GHz)	Antenna dimension (mm^3^)	Antenna type & Material	No of elements	Peak Gain (dBi)	Impedance bandwidth (MHz)	VSWR	Uniqueness in the Technique used	Polarization
^7^	2.4	43 × 40 × 1.6	Microstrip on FR-4	1	3.59	75	1.8	monostatic with dielectric variation for measuring S_11_	Linear
^9^	2.45	120 × 110** × **15	waveguide Horn antenna	1	4.7	68	1.6	Fat grafting technique to improve hyperthermia system. S parameter at the air-phantom interface is used	Left Hand Circularly polarized
^19^	2.5–6	33 × 50 × 1.6	Monopole on FR4	8	3.6	101	1.4	Differential imaging between two slots	Elliptical and linear
^22^	3–6	53 × 27.5** × **23 (Circular ring of 150 mm dia)	Dielectric ceramic-filled double-ridged waveguide	8	4.8	88	1.75	8 elements are placed on a circular ring capable of moving up and down with rotation to check return loss	Right hand circularly polarized
^28^	2–5	68 × 48 × 10	Spiral antenna on Kapton polyimide	16	3.4	152	1.3	copper and liquid based split-ring resonator for frequency tunability	Linearly polarized
^32^	2–5	65 × 40 × 5	Monopole on Kapton polyimide	16	4.2	164	1.4	Matching network with PIN diodes and varactors that can be electronically reconfigured	Linearly polarized
Proposed work (Dual mode using CMA)	2.5–5.3	50 × 50 × 1.6	Dual mode using CMA (PIN diode), FR4	1	5.31	110 (2420–2520), 170 (3410–3550)	1.2	Defected V-slotted ground to achieve RHCP and frequency diversity	Linear and Right hand circularly polarized

## Conclusion

The first section introduces a dual-mode dual-polarized antenna configuration that is based on characteristic mode analysis. Additionally, it presents a broadband dual-polarized antenna design for 5G communications networks, which utilizes a ring-shaped radiator. An extended elliptical patch is produced from the circular one, resulting in dual resonance by mode separation. The antenna operates within the 2.5 GHz IRNSS band, with a bandwidth of 110 MHz, and also covers the S-band, which has an impedance bandwidth of 170 MHz. The system utilizes a coaxial probe feed to achieve both vertical and horizontal polarization over two frequency bands. The working of the antenna is analyzed using CMA and current distribution. The antenna provides a satisfactory level of gain in both the operational frequency range and maintains a consistent radiation pattern. This simple antenna is a suitable option for applications operating below 6 GHz. In the second portion, a broadband dual-polarized antenna is developed utilizing a ring-shaped radiator. By using a PIN diode, the radiation properties can be altered, enabling the transition from linear polarization (LP) to right-hand circular polarization (RHCP). This prototype demonstrates a impedance bandwidth of 3.2 GHz when the diode is in the ON state and 1.1 GHz when the diode is in the OFF state. The maximum gain achieved in this scenario is 6.5 dBic in circular polarization. The results indicate that the fabricated antenna exhibits favorable radiation characteristics and has the potential to achieve exceptional performance in the 5G mid band for various applications.

## Data Availability

All data generated or analyzed during this study are included in this published article.
